# A randomized, double-blind, placebo-controlled phase II trial to explore the effects of a GABA_A_-α5 NAM (basmisanil) on intellectual disability associated with Down syndrome

**DOI:** 10.1186/s11689-022-09418-0

**Published:** 2022-02-05

**Authors:** Celia Goeldner, Priya S. Kishnani, Brian G. Skotko, Julian Lirio Casero, Joerg F. Hipp, Michael Derks, Maria-Clemencia Hernandez, Omar Khwaja, Sian Lennon-Chrimes, Jana Noeldeke, Sabine Pellicer, Lisa Squassante, Jeannie Visootsak, Christoph Wandel, Paulo Fontoura, Xavier Liogier d’Ardhuy, Clematis Study Group, Rafael De La Torre Fornell, Paul Glue, Julie Hoover-Fong, Sonja Uhlmann, Jorge Malagón Valdez, Andrew Marshall, Federico Martinón-Torres, Lorenzo Redondo-Collazo, Carmen Rodriguez-Tenreiro, Valeria Marquez Chin, Adriana G. Michel Reynoso, Ed A. Mitchell, Rebecca F. Slykerman, Trecia Wouldes, Sarah Loveday, Fernando Moldenhauer, Ramon Novell, Cesar Ochoa, Michael S. Rafii, Anne-Sophie Rebillat, Damien Sanlaville, Pierre Sarda, Rohit Shankar, Margaret Pulsifer, Casey L. Evans, Alexandra M. Silva, Mary Ellen McDonough, Maria Stanley, Lindsay M. McCary, Stefano Vicari, William Wilcox, Giuseppe Zampino, Alessandro Zuddas

**Affiliations:** 1 Neuroscience and Rare Diseases Discovery and Translational Area, Roche Pharmaceutical Research and Early Development, Roche Innovation Center Basel, Grenzacherstrasse 124, 4070 Basel, Switzerland; 2 Duke Clinical Research Institute, Durham, NC 27710 USA; 3 Down Syndrome Program, Division of Medical Genetics and Metabolism, Department of Pediatrics, Massachusetts General Hospital, Medical Genetics, Boston, MA 02114 USA; 4 Department of Pediatrics, Harvard Medical School, Boston, MA USA; 5 Hospital Infantil Universitario Niño Jesus, Pediatria Social, 28009 Madrid, Spain; 6 Pharmaceutical Sciences, Roche Pharmaceutical Research and Early Development, Roche Innovation Center Welwyn, Welwyn Garden City, UK; 7 Current affiliation: VectivBio AG, Basel, Switzerland; 8 Neuroscience and Rare Diseases Discovery and Translational Area, Roche Pharmaceutical Research and Early Development, Roche Innovation Center New York, New York, USA; 9 Current affiliation: Novartis Gene Therapies, New York, USA; 10 Pharmaceutical Sciences, Roche Pharmaceutical Research and Early Development, Roche Innovation Center Basel, Basel, Switzerland; 11 Roche Neuroscience Product Development, Basel, Switzerland; 12 Current affiliation: Loulou Foundation, London, UK

**Keywords:** Down syndrome, GABA_A_-α5, Cognition, Adaptive behavior, EEG

## Abstract

**Background:**

There are currently no pharmacological therapies to address the intellectual disability associated with Down syndrome. Excitatory/inhibitory imbalance has been hypothesized to contribute to impairments in cognitive functioning in Down syndrome. Negative modulation of the GABA_A_-α5 receptor is proposed as a mechanism to attenuate GABAergic function and restore the excitatory/inhibitory balance.

**Methods:**

Basmisanil, a selective GABA_A_-α5 negative allosteric modulator, was evaluated at 120 mg or 240 mg BID (80 or 160 mg for 12–13 years) in a 6-month, randomized, double-blind, placebo-controlled phase II trial (Clematis) for efficacy and safety in adolescents and young adults with Down syndrome. The primary endpoint was based on a composite analysis of working memory (Repeatable Battery for the Assessment of Neuropsychological Scale [RBANS]) and independent functioning and adaptive behavior (Vineland Adaptive Behavior Scales [VABS-II] or the Clinical Global Impression-Improvement [CGI-I]). Secondary measures included the Behavior Rating Inventory of Executive Functioning-Preschool (BRIEF-P), Clinical Evaluation of Language Fundamentals (CELF-4), and Pediatric Quality of Life Inventory (Peds-QL). EEG was conducted for safety monitoring and quantitatively analyzed in adolescents.

**Results:**

Basmisanil was safe and well-tolerated; the frequency and nature of adverse events were similar in basmisanil and placebo arms. EEG revealed treatment-related changes in spectral power (increase in low ~ 4-Hz and decrease in high ~ 20-Hz frequencies) providing evidence of functional target engagement. All treatment arms had a similar proportion of participants showing above-threshold improvement on the primary composite endpoint, evaluating concomitant responses in cognition and independent functioning (29% in placebo, 20% in low dose, and 25% in high dose). Further analysis of the individual measures contributing to the primary endpoint revealed no difference between placebo and basmisanil-treated groups in either adolescents or adults. There were also no differences across the secondary endpoints assessing changes in executive function, language, or quality of life.

**Conclusions:**

Basmisanil did not meet the primary efficacy objective of concomitant improvement on cognition and adaptive functioning after 6 months of treatment, despite evidence for target engagement. This study provides key learnings for future clinical trials in Down syndrome.

**Trial registration:**

The study was registered on December 31, 2013, at clinicaltrials.gov as NCT02024789.

**Supplementary Information:**

The online version contains supplementary material available at 10.1186/s11689-022-09418-0.

## Background

Down syndrome (DS), the triplication of whole or part of chromosome 21, is the most common identifiable cause of intellectual disability with an incidence of 1 in 650 to 1 in 1000 live births per year worldwide [1, 2]. Among several co-occurring conditions, DS is associated with a unique cognitive and adaptive behavior profile [3, 4], which is of primary concern to many caregivers. Since more individuals with DS are active members of the community due to increased life expectancy, improving functional potential through development of pharmacotherapies may address these unmet needs. There is currently no therapeutic option available to treat the associated intellectual disability.

Although the etiology of the cognitive disability in people with DS remains unclear, cellular and anatomical abnormalities in the prenatal and perinatal forebrain and cerebellum suggest that early brain development is altered in individuals with DS [5–7]. Similar brain abnormalities have been described in mouse models of DS, such as the Ts65Dn which is the best characterized model [8–10]. Studies have suggested that the major functional defect in the postnatal Ts65Dn brain may be an imbalance between excitatory and inhibitory circuits [11–13]. Chronic treatment with selective GABA_A_-α5 negative allosteric modulators (NAMs)—such as α5IA [14], RO4938581 [15], and basmisanil [16]—improved synaptic plasticity and rescued cognitive and behavioral deficits in Ts65Dn mice, without inducing anxiety or convulsions, side effects observed with non-selective GABA_A_ NAMs [17, 18]. Inhibition of GABA_A_-α5 receptors may represent an attractive mechanism to enhance cognition in individuals with DS.

Basmisanil (RO5186582, RG1662) is a potent NAM, which combines both binding and functional selectivity at GABA_A_-α5 subunit-containing receptors and has been shown to improve cognition in rats and monkeys [19]. GABA_A_-α5 hippocampal receptor occupancy between 30–65% was required for efficacy in preclinical studies [16, 19]. Basmisanil has shown a favorable safety and tolerability profile over a broad range of doses in healthy volunteer studies (BP25611 [ClinicalTrials.gov: NCT01667367], WP28214 [NCT01684891]; BP25129 [EudraCT: 2009-016097-33], WP25366 [2010-021554-19]), and in adults with DS (BP25543 [NCT01436955], BP25611 [NCT01667367]).

Given the absence of any effective therapy for the intellectual disability associated with DS, the supportive 5-week safety and tolerability profile established in individuals aged 18–30 years with DS (BP25543; Additional file 1) and the potential added benefit of earlier intervention, we aimed to assess the efficacy of extended basmisanil dosing on cognition and adaptive behavior in both adolescents and young adults with DS.

## Methods

### Participants

Male and female participants (12–30 years) with DS (standard trisomy 21, Robertsonian translocation, isochromosome 21, with reciprocal translocation, or mosaicism) were included. Minimum verbal abilities were required to participate in the study, as defined by a minimum raw score of 7 for adults, or 4 for adolescents, on the Clinical Evaluation of Language Fundamentals Preschool-2 (CELF-P) Word Classes subtests [20]. The IQ of participants was assessed at baseline only using the non-verbal Leiter 3 test [21].

Individuals with a diagnosis of autism spectrum disorder, major depressive disorder, a history of infantile spasms or epileptic encephalopathy, or a history of seizures within 2 years prior to the screening visit were not included in the trial. Participants consented or assented to participate, and written informed consent was obtained from their caregiver.

### Study design

BP27832 (Clematis) was a randomized, double-blind, placebo-controlled, multi-country phase II study to investigate the efficacy and safety of basmisanil in adults (18–30 years) and adolescents (12–17 years) with DS (Additional file 2). The study was registered on December 31, 2013, at clinicaltrials.gov as NCT02024789, approved by local ethics committees, and conducted in accordance with the principles of the “Declaration of Helsinki” and Good Clinical Practice. A Roche-independent safety committee was responsible for the monitoring of safety data on a regular basis.

Eligible participants were randomized in a 1:1:1 ratio to receive either tablets of placebo, low or high dose of basmisanil, twice daily (BID) over 6 months (26 weeks). The low dose of basmisanil was 120 mg and the high dose was 240 mg, except for participants below 14 years where the low dose was age-adjusted to 80 mg and the high dose to 160 mg. Dose selection was based on an integrated evaluation of pharmacokinetics (PK), pharmacodynamics, PET (BP25611; Additional file 1), and safety data from prior clinical studies with basmisanil in healthy volunteers and adults with DS, coupled with preclinical safety and efficacy data. The aim was to have two effective dosing regimens: the low dose targeted exposures that would result in receptor occupancy in all individuals above a minimum threshold of 60% expected to be required for efficacy based on preclinical models of DS [19]; the high dose was selected to reach exposures predicted to maintain receptor occupancy above a near-maximal threshold (> 90%).

### Primary and secondary efficacy

Efficacy assessments were performed at baseline and after 3 and 6 months of treatment. The primary efficacy analysis assessed the proportion of participants who showed improvement above pre-defined thresholds (i.e., above-threshold improvement) on a composite endpoint, concomitantly evaluating cognition and adaptive functioning, after 6 months of treatment. Above-threshold improvement on the composite endpoint was defined as (1) a relevant increase in raw scores from baseline in at least two out of three tasks from the Repeatable Battery for the Assessment of Neuropsychological Status ([RBANS]; at least 2 points for list learning and 1 point for list recognition and list recall); and (2) either an increase from baseline in the Vineland Adaptive Behavior Scales-II (VABS-II) composite standard score of ≥ 7 or a Down syndrome-specific Clinical Global Impression-Improvement (DS-CGI-I) score of ≤ 3 (minimally improved). The DS-CGI-I evaluation was based on scoring DS-specific anchors: communication/speech, activities of daily living, social functioning, and stubbornness/non-compliance (Additional file 3). The RBANS thresholds were identified based on the variability of each endpoint observed at baseline in the observational study, conducted in a comparable population in terms of average age and IQ [22]. They correspond to an effect size of approximately 0.3, i.e., 30% of the standard deviation observed in baseline raw scores for each task. These RBANS thresholds were then discussed in an advisory board meeting, with clinicians and clinical research experts in neurodevelopmental disorders and DS, to qualitatively assess the clinical meaningfulness of these changes. The selected thresholds were considered adequate across the age range if concomitant improvements could be observed on global functioning measures of established clinical relevance such as the CGI or the VABS. The secondary efficacy analyses evaluated change from baseline scores on each of the individual measures contributing to the composite endpoint, (RBANS learning, recognition and recall tasks raw scores; VABS-II composite standard score; DS-CGI-I score). Treatment effects on VABS-II domain standard scores (communication, daily living skills, and socialization), language (word classes tasks of Clinical Evaluation of Language Fundamentals-version 4 [CELF-4] raw scores), executive function (Behavior Rating Inventory of Executive Function Preschool [BRIEF-P] raw scores), and global quality of life (Pediatric Quality of Life Inventory [PedsQL] raw scores) were also evaluated.

### Statistical analysis of efficacy endpoints

Fifty subjects per treatment group provide a power of 80% to detect a difference between each active dose and placebo when the frequency of participants with above-threshold improvement is 30% on active dose and 5% on placebo. This calculation was based on the two-sided *χ*^2^ test with continuity correction and significance declared at the two-sided 2.5% level to maintain the overall 5% level study-wise (as per Bonferroni adjustment for multiple comparisons).

The proportion of participants with above-threshold improvement was analyzed by means of a logistic regression model. This included treatment and visit and treatment by visit interaction, age, sex, and IQ at baseline as covariates, participant as repeated effect. The selected covariates were defined a priori in a statistical analysis plan, as sex and age may have an impact on drug pharmacokinetic properties, and age and IQ are expected to influence cognition, language, and adaptive behavior in individuals with DS. For all endpoints normally distributed a mixed model analysis of variance was applied to change from baseline scores, where applicable, with baseline, age, sex, and IQ at baseline as covariates, treatment and treatment by visit interaction, with visit as repeated measurements and participant as random. Inferential findings are provided for descriptive purposes only and without any confirmatory meaning. Multiple endpoints and multiple treatment comparisons were analyzed; however, due to the exploratory nature of the study, multiplicity was not statistically adjusted for, and the risk of false positive results should be taken into consideration in the interpretation of the results.

### Pharmacokinetic assessments

Blood samples were collected for determination of plasma concentrations of basmisanil. Concentrations were measured by a specific liquid chromatography-mass spectrometry/mass spectrometry method. The following time points were included prior to dosing to assess trough concentrations of basmisanil at weeks 2, 6, and 12.

### Safety assessments

Safety surveillance of participants included adverse event (AE) reporting, physical examinations, vital signs including 12-lead ECG recordings, clinical chemistry, hematology, and urinalyses. Comorbidities were monitored, such as ADHD (Conner’s questionnaire); sleep problems (Children’s sleep habits questionnaire); anxiety and depression (ADAMS questionnaire). As per regulatory guidance, suicidality monitoring was implemented using the pediatric and adult C-SSRS version.

### EEG assessments

EEG recordings were primarily included to monitor the emergence of epileptiform abnormalities in adolescents and participants with a medical history of epilepsy, to confirm the favorable safety profile of basmisanil previously established in adults with DS (without a medical history of epilepsy, study BP25543). A 30-min EEG recording was performed at baseline (pre-dose), week 2, and week 20. Recordings from adolescents were used for the exploratory quantitative EEG analyses reported here. The exploratory quantitative analyses of the EEG data were restricted to spectral power, which provides a macroscopic measure of synchronized neuronal activity. No assumptions were made about spectral or spatial properties of possible treatment effects. The statistical analysis accounted for multiple comparisons across frequencies and electrodes using a cluster randomization approach.

To test for a PK-PD relationship we performed non-parametric correlations (Spearman rank correlation; one-tailed test, i.e., testing for a positive correlation for theta-band power and negative correlation for beta-band power) between individual measured trough exposure levels and theta and beta-band EEG power from the identified clusters both measured at week 2. Although the EEG was recorded 4–5 h post administration, and the PK sample before administration, the measured trough concentrations at steady state are considered as a reasonable proxy for the individual basmisanil concentration at the time of the EEG recording. For this analysis we used all dosed participants but only included participants with a PK sample and an EEG recording at week 2 (*n* = 37, low dose: *n* = 14, high dose: *n* = 23). Full details on the EEG acquisition and analysis can be found in Additional file 4.

## Results

### Enrollment

Between May 5, 2014, and October 1, 2015, 170 participants were randomized across 30 sites. For adults, the majority were recruited at US (60%), French (20%), and Spanish (13%) sites. For adolescents, the majority were recruited at Spanish (42%) and US (31%) sites.

**Fig. 1:**
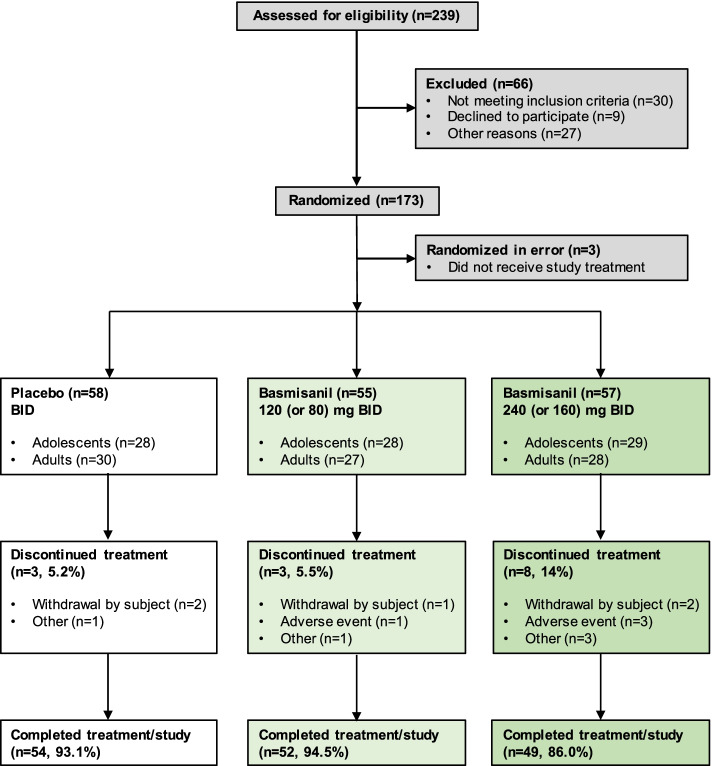
Participant disposition (CONSORT diagram)

A total of 155 participants (91%) completed the study and were included in the analysis (Fig. 1). The proportion of participants who discontinued study medication prematurely was higher in the high-dose arm (8/57, 14%) than in placebo (3/58, 5.2%) and low-dose (3/55, 5.5%) arms. Withdrawals were mostly driven by non-safety-related reasons (placebo: 3/3; low dose: 2/3; high dose: 5/8). The majority of deviations to the protocol were assessments being performed outside the defined visit window due to scheduling issues. Seven participants were excluded from the efficacy analysis population (six did not meet CELF-P inclusion criterion, one had < 80% compliance rate to study medication).

Participants’ demographics and baseline characteristics were similar across arms (Table 1). Approximately two-thirds of the study population were taking concomitant therapies; the most prescribed treatments across all groups were analgesics/non-steroidal anti-inflammatory drugs (17–31%), corticosteroids (3–19%), and penicillin drugs (9–18%).

**Table 1:** Baseline characteristics

	Placebo	Basmisanil	
		120 mg (80 mg)	240 mg (160 mg)
	*n* = 58	*n* = 55	*n* = 57
**Age (years)**			
Mean ± SD	18.7 ± 5.2	18.3 ± 4.9	18.7 ± 5.4
Median	18.0	17.0	17.0
Minimum to maximum	12–30	12–28	12–29
12–17 years: *n* (%)	28 (48%)	28 (51%)	29 (51%)
18–30 years: *n* (%)	30 (52%)	27 (49%)	28 (49%)
**Sex**			
Males: *n* (%)	33 (57%)	32 (58%)	38 (67%)
Females: *n* (%)	25 (43%)	23 (42%)	19 (33%)
**Ethnicity**			
Hispanic or Latino: *n* (%)	11 (19.0%)	13 (23.6%)	10 (17.5%)
Not Hispanic or Latino: *n* (%)	38 (65.5%)	35 (63.6%)	39 (68.4%)
Unknown: n (%)	9 (15.5%)	7 (12.7%)	8 (14.0%)
**Race:** *n* (%)			
American Indian or Alaska Native	1 (1.7%)	0	0
Asian	1 (1.7%)	1 (1.8%)	0
Black or African American	0	2 (3.6%)	0
Multiple: White/Asian	2 (3.4%)	0	0
White	45 (77.6%)	44 (80.0%)	49 (86.0%)
Unknown	9 (15.5%)	8 (14.5%)	8 (14.0%)
**Formulation**^a^			
Granules: *n* (%)	10 (17%)	10 (18%)	12 (21%)
Tablets: *n* (%)	48 (83%)	45 (82%)	45 (79%)
**CGI-severity**			
Mean ± SD	3.7 ± 1.0	3.8 ± 0.9	3.9 ± 0.9
Median	4.0	4.0	4.0
Minimum to maximum	1–5	1–5	1–6
≤ 3: *n* (%)	18 (31%)	16 (31%)	12 (22%)
> 3: *n* (%)	40 (69%)	36 (69%)	43 (78%)
**CELF-4 (word classes 1):** mean ± SD			
Receptive	15.2 ± 4.3	15.2 ± 3.9	14.7 ± 4.9
Expressive	9.7 ± 5.4	9.6 ± 4.2	9.8 ± 5.1
**CELF-4 (word classes 2):** mean ± SD			
Receptive	3.5 ± 4.1	2.98 ± 3.4	2.8 ± 3.2
Expressive	1.8 ± 2.3	1.4 ± 1.7	1.5 ± 1.9
**Anxiety/mood (ADAMS):** mean ± SD			
Depressed mood	2.5 ± 2.9	2.1 ± 2.0	2.5 ± 3.1
Anxiety	2.6 ± 2.3	2.5 ± 2.5	2.6 ± 2.9
Manic/hyperactive	3.2 ± 2.4	4.0 ± 3.1	3.1 ± 2.8
Obsessive/compulsive	1.9 ± 2.0	1.9 ± 1.8	1.7 ± 1.8
Social avoidance	3.8 ± 3.9	4.3 ± 3.7	3.8 ± 3.9
**IQ**^**b**^			
Mean ± SD	52.8 ± 13.6	55.2 ± 14.6	55.6 ± 13.9
Median	49	53	57
Minimum to maximum	32–93	32–93	32–80

*Abbreviations*: *ADAMS* Anxiety, Depression and Mood Abnormalities, *CELF* Clinical Evaluation of Language Fundamentals, *CGI* Clinical Global Impression, *SD* standard deviation

^a^A granule formulation was available for individuals with difficulties swallowing tablets (assessed in comparative bioavailability study WP28978 [NCT02194244])

^b^IQ assessed by Leiter International Performance Scale-revised: a non-verbal intelligence test

### Primary efficacy: composite endpoint analysis at 6 months

**Fig. 2:**
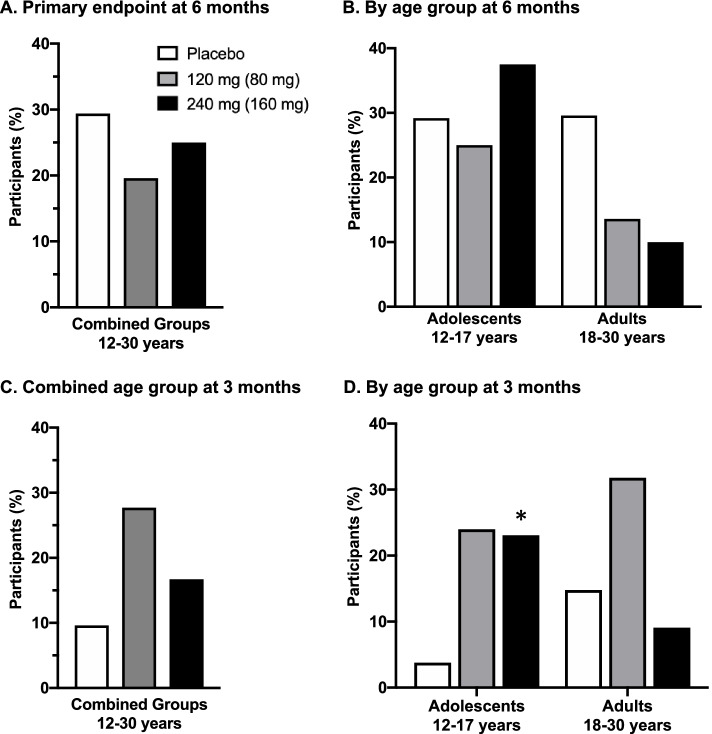
Percent of participants with above-threshold improvement on the composite endpoint. **A** Primary efficacy endpoint after 6 months of treatment. Percent of participants with above-threshold improvement: **B** by age group (adolescents, adults) after 6 months of treatment; **C** combined age group after 3 months of treatment; **D** by age group (adolescents, adults) after 3 months of treatment. Above-threshold improvement on the composite endpoint was defined as having (1) a relevant increase in raw scores from baseline in at least two out of three tasks from the Repeatable Battery for the Assessment of Neuropsychological Status ([RBANS]; ≥ 2 points for list learning, ≥ 1 point for list recognition, ≥ 1 point for list recall); and (2) either an increase from baseline in the Vineland Adaptive Behavior Scales-II (VABS II) composite score of ≥ 7 or a Down syndrome-specific Clinical Global Impression-Improvement (DS-CGI-I) ≤ 3 (minimally improved). Efficacy assessments were performed at baseline and after 3 and 6 months of treatment. Statistics: *p < 0.05 vs. placebo-treated group

The findings of the study indicate lack of treatment effects on the primary endpoint. The proportion of participants with above-threshold improvement on the composite endpoint at 6 months was not different between basmisanil-treated groups and placebo (*p* = 0.262; Fig. 2A). Subgroup analyses by age (Fig. 2B), or by sex, language (English-speaking countries, Rest of the World), functioning level (IQ < 50, ≥ 50), and expressive abilities based on CELF−P score at screening (adolescents < 7 or ≥ 7; adults < 10 or ≥ 10) also showed lack of a treatment effect (data not shown).

At 3 months there was no statistically significant difference in improvement overall (Fig. 2C), however, in the adolescents (Fig. 2D) a higher proportion of participants with above-threshold improvement was observed in both basmisanil-treated groups compared to the placebo group, with a nominal *p*-value of *p* = 0.043 at the high dose (low dose, nominal *p*-value: *p* = 0.063).

Additionally, no differences in the proportion of participants with above-threshold improvements were detected between placebo and basmisanil-treated groups on any of the individual components of the composite endpoint (RBANS, VABS-II and DS-CGI-I; Additional file 5).

### Secondary efficacy outcome measures

There were no statistically significant differences between placebo and basmisanil-treated groups in secondary outcome measures evaluating changes from baseline (Table 2) in cognition (RBANS), adaptive behavior (VABS-II composite), language (CELF-4), executive function (BRIEF-P), or global quality of life (PedsQL). In both basmisanil and placebo groups, small improvements were observed in RBANS list learning, BRIEF-P and PedsQL (Table 2), as well as in the VABS-II domain scores of socialization, communication, and daily living skills (Additional file 6). Nearly all participants were able to reach CELF-4 Word Class 2 level and no improvements in receptive or expressive language abilities were observed over 6 months across treatment arms (Table 2).

**Table 2:** Change from baseline scores at 3 and 6 months

Assessment	Time point (month)	Placebo		120 (80) mg			240 (160) mg		
		Mean ± SD	***n***	Mean ± SD	***n***	***p***	Mean ± SD	***n***	***p***
**RBANS**									
List learning	3	1.5 ± 5.2	54	2.3 ± 5.4	47	0.69	1.0 ± 5.12	48	0.86
	6	3.1 ± 6.1	51	2.7 ± 6.2	47	0.56	2.4 ± 4.8	44	0.76
List recall	3	0.5 ± 2.8	53	0.2 ± 2.2	47	0.49	0.4 ± 2.5	48	0.98
	6	0.3 ± 2.4	51	0.2 ± 3.1	47	0.98	-0.1 ± 2.4	44	0.83
List recognition	3	0.0 ± 2.9	53	1.1 ± 2.3	47	0.09	0.8 ± 4.2	48	0.26
	6	1.2 ± 3.2	51	1.4 ± 3.1	47	0.75	1.8 ± 3.9	44	0.29
**VABS-II**									
Composite score	3	1.6 ± 5.0	53	0.98 ± 4.6	46	0.60	1.02 ± 3.7	47	0.62
	6	2.4 ± 10.2	50	2.0 ± 4.02	46	0.79	2.02 ± 4.6	43	0.72
**CELF-4 (word classes 1)**									
Receptive	3	− 0.2 ± 3.7	54	− 0.5 ± 4.1	47	0.74	1.1 ± 2.8	48	0.09
	6	0.8 ± 3.6	51	− 0.07 ± 3.4	46	0.28	1.5 ± 3.6	44	0.31
Expressive	3	0.5 ± 3.8	54	0.6 ± 3.9	47	0.79	1.2 ± 3.2	48	0.22
	6	0.9 ± 3.1	51	0.3 ± 3.8	46	0.34	1.3 ± 3.1	44	0.54
**CELF-4 (word classes 2)**									
Receptive	3	0.1 ± 2.1	51	− 0.2 ± 2.0	43	0.23	0.0 ± 2.1	43	0.60
	6	− 0.3 ± 3.6	47	0.0 ± 3.1	41	0.99	0.2 ± 2.3	40	0.52
Expressive	3	0.2 ± 1.5	51	− 0.07 ± 1.1	43	0.14	0.2 ± 1.5	43	0.99
	6	0.1 ± 2.4	47	− 0.07 ± 1.4	41	0.41	0.2 ± 1.6	40	0.78
**BRIEF-P**^**a**^									
Global executive composite	3	− 4.1 ± 12.3	53	− 6.6 ± 12.7	47	0.48	− 5.2 ± 11.6	48	0.75
	6	− 4.1 ± 12.2	51	− 7.8 ± 12.6	46	0.16	− 7.9 ± 12.7	42	0.10
**PedsQL**									
Total scale score	3	0.9 ± 14.0	54	2.7 ± 15.3	48	0.75	3.9 ± 12.1	45	0.19
	6	1.7 ± 12.7	47	5.6 ± 12.5	46	0.31	3.5 ± 9.7	41	0.46

See Additional file 12 for “change from baseline” scores by age group and time point

*Abbreviations*: *BRIEF-P* Behavior Rating Inventory of Executive Function-Preschool, *CELF* Clinical Evaluation of Language Fundamentals, *PedsQL* Pediatric Quality of Life Inventory, *RBANS* Repeatable Battery for the Assessment of Neuropsychological Status, *SD* standard deviation, *VABS*-II Vineland Adaptive Behavior Scales-II

^a^Negative change = improvement

### Exploratory qEEG in adolescents

**Fig. 3:**
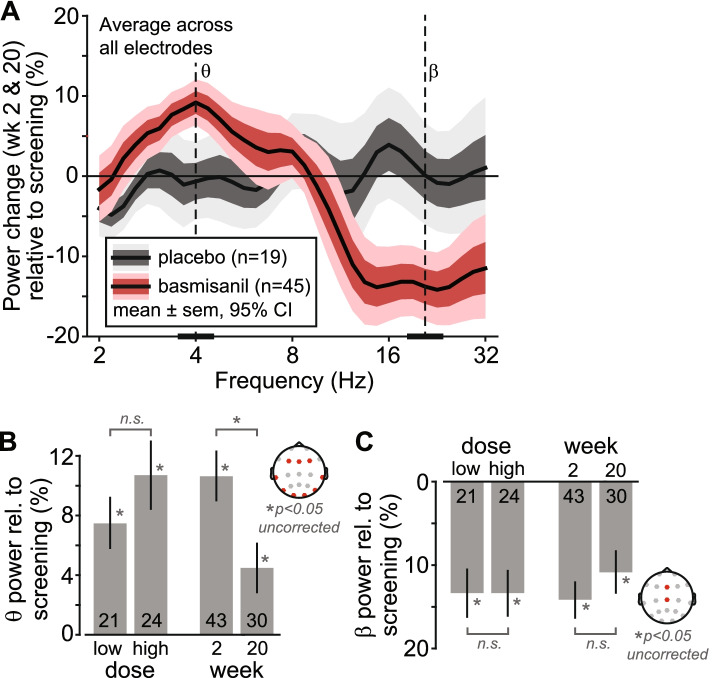
Quantitative EEG. **A** Change in EEG spectral power (average across week 2 and week 20 visits relative to baseline) for dosed (red) and the placebo (gray) groups. **B**, **C** Effects of assessment time-point (week 2 vs. week 20) and dose (low dose vs. high dose) for signal power extracted from the centers of the clusters identified in 1.2.3 (theta cluster, frequency range [3 bins]: ~ 3.5–4.5 Hz, electrodes: F3, Fz, F4, T7, T8, P7, P8, O1, O2; beta cluster, frequency range [3 bins]: ~ 19–22.5 Hz, electrodes: Fz, Cz). The top plots indicate the electrodes used for each extraction of signal power. The numbers at the base of the bars indicate the number of participants entering the analyses

The baseline EEG power spectrum was characterized by a marked absence of an alpha peak, which is the most prominent feature of typical developing individuals, and exhibited a prominent peak in the theta frequency range around 4 Hz (Additional file 7: panel B). In response to basmisanil, relative spectral power at lower frequencies (~ 4-Hz, theta-frequency range) increased while relative power at higher frequencies (~ 20-Hz, beta-frequency range) decreased compared to baseline, but spectral power remained unchanged for placebo (Fig. 3A). Absolute power also revealed an increase in the theta- and decrease in the beta-frequency range in response to basmisanil (Additional file 7: panel G). These qualitative observations were confirmed by statistical analysis using cluster-randomization that accounted for multiple testing across all electrodes (*n* = 19) and frequencies (2–32 Hz). The analysis identified two clusters, i.e., differences between the combined dose groups and placebo that extended across frequencies and electrodes. A “positive cluster” in the theta-frequency range (power increase for dose groups relative to placebo, *p* = 0.022) and a “negative cluster” in the beta-frequency range (power decrease for dose groups relative to placebo, *p* = 0.0007; Additional file 7: panel B-E).

For further characterization of the theta- and beta-band effects we extracted signal power from the “centers” of these clusters as pharmacodynamics parameter (Fig. 3B, C): power change from baseline (mean ± sem) and effect size for theta: 9.2±1.46%, d’ = 0.94; and beta: − 13.4±1.92%, d’ = − 1.04. These values are subject to a positive selection bias and should be considered as upper bounds.

There was no difference between the low or high dose (theta: *p* = 0.27, beta: *p* = 0.99; Fig. 3B, C). The EEG effects appeared weaker for week 20 compared to week 2. The decline was significant for the theta-band (*p* = 0.041, uncorrected for multiple testing) but not for the beta band (*p* = 0.27).

Neither the theta-band nor the beta-band EEG pharmacodynamic effects correlated with exposure (theta: rho = 0.217, *p* = 0.1; beta: rho = − 0.168, *p* = 0.16; *n* = 37). Numerically, the correlations were in the expected direction (positive for theta power, negative for beta) but lacked significance.

### Pharmacokinetics

Comparable trough exposures were observed for the high dose between adults and adolescents aged 14–17 years (Additional file 8, Table 1). The low dose in adolescents aged 12–17 and the high dose in 12–13-year-olds resulted in lower exposures than adults. Overall, comparable average trough exposures were observed between adults and all adolescents (12–17 years) for the age-adjusted high doses, while differences were noted for the age-adjusted low doses, which resulted in slightly lower exposures in adolescents. Overall, the measured trough concentrations remained stable (Additional file 8, Table 3) and adherence to study medication was high throughout the study.

#### Predicted receptor occupancy

The low and high doses provided high predicted receptor occupancies of 83% and 92%, respectively, in the overall population (Additional file 8, Table 2), indicating a lack of separation of the two selected doses. At the high dose, the average predicted receptor occupancy at trough was comparable between adolescents (92%) and adults (93%). At the low dose, lower receptor occupancy was noted in adolescents (77%) compared to adults (87%) (Additional file 8, Table 1). There were no relevant differences in exposure or receptor occupancy between participants with and without above-threshold improvement (data not shown).

### Safety

The frequency and nature of AEs were similarly distributed among placebo and basmisanil-treated participants (Table 3). There were no treatment-emergent epileptiform abnormalities noted during EEG monitoring in any participant.

**Table 3:** Adverse events by treatment group

**Adverse events (in more than 5% of participants)**	**Placebo**		**120 mg (80 mg)**		**240 mg (160 mg)**	
	***n***	**%**	***n***	**%**	***n***	**%**
Infections and infestations	32	55.2	24	43.6	24	42.1
Gastrointestinal disorders	18	31.0	12	21.8	15	26.3
Nervous system disorders	14	24.1	13	23.6	13	22.8
Investigations	12	20.7	5	9.1	6	10.5
Skin and subcutaneous tissue disorders	8	13.8	6	10.9	6	10.5
Psychiatric disorders	5	8.6	6	10.9	8	14.0
General disorders and administration site conditions	5	8.6	7	12.7	6	10.5
Respiratory, thoracic, and mediastinal disorders	4	6.9	7	12.7	2	3.5
Musculoskeletal and connective tissue disorders	2	3.4	5	9.1	5	8.8
Eye disorders	4	6.9	3	5.5	1	1.8
Injury, poisoning and procedural complications	0	0	4	7.3	3	5.3
**Serious adverse events**	***n***		***n***		***n***	
Suicidal ideation	1				1	
Altered state of consciousness			1			
Skin laceration			1			
Salmonellosis					1	

Five serious AEs, reported in five participants, were considered not related to treatment (Table 3), and one event (altered state of consciousness) led to study withdrawal. In addition, non-serious AEs in three participants resulted in study withdrawal. Overall, the number of participants withdrawn from treatment due to AEs was low and did not point to a particular AE pattern (high-dose group [*n* = 3]: combination of “headache, nausea, vomiting” with treatment stop on study day 113; “sleep apnea syndrome” with treatment stop on day 45; and “nightmares” with treatment stop on day 98; low dose group [*n* = 1]: “altered state of consciousness” with treatment stop on day 60; placebo group: no subject withdrawn due to AE).

Vital sign monitoring did not reveal changes in heart rate and blood pressure (Additional file 9). QTcF analyses in ECG monitoring did not reveal an alert of relevant QTc prolongation (Additional file 10). Monitoring of co-occurring symptoms did not reveal notable changes as summarized in Additional file 11 and there was no signal on suicidality risk associated with basmisanil treatment.

## Discussion

Clematis was the first phase II trial performed in the DS population with a compound specifically designed to address excessive inhibition in limbic brain areas, hypothesized to contribute to the intellectual disability associated with DS [14, 15]. Overall, the findings of this study indicate that 6 months of treatment with the GABA_A_-α5 receptor NAM basmisanil was safe and well-tolerated, but did not reveal any effects of treatment on primary and secondary measures of efficacy, suggesting it did not improve cognition or functioning in adults and adolescents with DS. The observed basmisanil exposures were stable and marginally lower in the adolescents. Although the exposures remained within the predicted range from the population PK model, both doses resulted in high average predicted receptor occupancy which did not clearly separate (low dose: 83% and high dose: 92%) and could thus be expected to be efficacious. The lack of differentiation between doses limits meaningful interpretations of dose-dependent treatment effects from both safety and efficacy perspectives in the overall population. In adolescents, there was a higher proportion of participants showing improvement on the primary endpoint after 3 months of treatment (nominal *p*-value < 0.05 at the high dose). This effect was not maintained after 6 months of treatment despite stable exposures and was not reflected in any of the secondary measures. The absence of differences in exposure-response relationships between participants with and without above-threshold improvements, across ages and doses (data not shown), corroborate a true lack of effect of basmisanil.

The primary endpoint was designed to capture potential improvements in intellectual functioning from multiple perspectives by combining direct measures of cognition (RBANS memory tasks), clinician ratings (DS-CGI-I), and caregiver-reported measure (VABS-II). These measures were selected based on their suitability for the population, reliability, stability over time, and feasibility of implementation, as previously determined in a 6-month observational study with a comparable study design and population [22, 23]. In the current study, the stability over time of most measures was not replicated; improvements were observed across placebo and treatment arms over 6 months on multiple variables including the VABS-II composite scores, DS-CGI-I, BRIEF-P, and PedsQL. The changes observed in this study, as compared to low natural improvement seen on the same measures in our previous non-interventional trial, may in part be attributed to the great anticipation of a potential therapeutic option among the DS community involved in this first large international clinical trial. The impact of treatment expectancy in clinical trials in pediatric neurodevelopmental disorders has been widely described, especially for caregiver-reported scales, and remains a key challenge for drug development [24, 25].

These changes were more pronounced in the adolescent population and are in line with published placebo response rates of 10–30% described in DS [26] and other neurodevelopmental conditions with intellectual disability, such as Fragile X syndrome or autism spectrum disorder [27]. In order to better control such effects, other researchers included regular cognitive training in both treated and placebo cohorts, with a run-in period, during a 6-month clinical trial in adults with DS [28].

The threshold for improvement on the primary composite endpoint combined improvements on RBANS memory tasks and global functioning on either the VABS-II or the DS-CGI-I. Because the DS-CGI-I anchors were mainly derived from the VABS-II domains, DS-CGI-I scores may not be independent of the caregiver perception captured by the VABS-II. The increases over time in VABS-II scores observed across groups may reflect treatment expectancy effects and directly (or indirectly via the DS-CGI-I) drive improvements on the primary endpoint. The composite endpoint is a multidimensional measure which increases the complexity of the analysis and interpretation and requires consistent effects to reach statistical significance. The choice of a composite endpoint, although a high bar objective, is unlikely to have masked effects as no beneficial treatment effects were detected on any of the individual components of the primary endpoint. Consistent with these findings, the analysis of secondary outcome measures did not show any beneficial effects of basmisanil over placebo after 6 months of treatment. Importantly, scores from the direct performance-based evaluations of cognition assessing memory (RBANS) and language (CELF), thought to be less sensitive to treatment expectancy bias, remained generally stable across age and treatment groups over the 6-month study duration, with the exception of the RBANS learning task. The small improvements observed in RBANS learning are in line with previous data from our observational study [22] and possibly reflect procedural learning due to repeated administration. Overall, this suggests that improvements in the placebo group are unlikely to have generally obscured treatment effects in the study. Of note, almost all participants were able to reach the second level of the CELF-4 and no floor effect was observed, suggesting that the CELF-4 word classes task can be used in future clinical trials with adults and adolescents with DS.

Exploratory quantitative analysis of EEGs recorded in adolescents was performed to test for effects on brain function. The absence of an alpha peak in the baseline EEG power spectrum is in line with previous findings in adults with DS reporting a shift to lower frequencies [29–31]. The basmisanil-induced pharmacodynamic effects, i.e., an increase in theta power (~4 Hz), and a decrease in beta power (~20 Hz) confirm the spectral signature of basmisanil that we have found previously in healthy volunteers [19] and demonstrate brain circuit engagement. In particular, EEG power in the beta frequency range has been linked to GABA_A_ function through pharmacology [32, 33], in rare genetic conditions involving CNVs [34, 35] and SNPs in GABA_A_ receptor genes [36, 37], and in modeling studies [38, 39]. Correlation analyses with individual basmisanil concentration did not reveal a significant dose dependence but were in the expected direction. The lack of a significance PK-PD relationship may relate to the overall high receptor occupancy (> 77% for all dose x age groups) where little dynamic range of the EEG PD effect may be expected, and to a limited sample size (Additional file 8: Table 1). In sum, the observed changes in the EEG in response to basmisanil can be considered evidence of functional target engagement.

While basmisanil exposure remained stable, the EEG effect in lower frequencies was weaker at week 20 compared to week 2, while remaining significantly higher than at baseline. The decrease in EEG power at lower frequencies may indicate compensatory or adaptive neuronal mechanisms that could result in tolerance. Tolerance is a well-described phenomenon for non-selective GABA_A_ receptor positive allosteric modulators after long-term use [40]. However, it is important to point out that the beta-band EEG effect, with an established link to GABA_A_ function did not significantly decline over time and no withdrawal effects were observed when the administration of basmisanil was stopped. Finally, there is no preclinical evidence suggesting that α5 subtype-selective compounds, such as basmisanil, lead to tolerance [41]. Tolerance to the effects of basmisanil is unlikely to underlie the lack of efficacy in this study.

Some study limitations should be noted. The detection of significant treatment effects of basmisanil may have been limited by the small sample size. Indeed a potential selection bias cannot be controlled for, albeit random treatment group assignment. Cognitive and behavioral measurements were not assessed during the first month of treatment; we are therefore unable to interpret potential improvements in relation to the early pharmacodynamic EEG changes observed. This would have also been helpful to interpret the trend observed after 3 months in adolescents, as well as the trends observed after 5 weeks of treatment on the RBANS tasks in a small exploratory phase IB trial in young adults with DS (BP25543; Additional file 1).

The detection of treatment effects of basmisanil may also have been hampered by the timing of the pharmacological intervention. Key brain development processes such as synaptogenesis and pruning [42] occur in early development before the age of 12 years. Modulation of GABA_A_-α5 receptors may therefore be more impactful during earlier stages of neural development, before long-term consequences of and adaptations to altered GABAergic inhibition have shaped brain function. Although our study did not demonstrate any evidence of age-dependent effects, a potential beneficial effect of basmisanil prior to the adolescent period cannot be fully excluded.

It is also conceivable that selective modulation of the GABA_A_-α5 receptor subtype or the maximal inhibitory effect of basmisanil on chloride channel current (~ 40%) [19] may not be sufficient to restore the excitatory/inhibitory imbalance hypothesized to underlie the cognitive profile of DS [15]. Alternatively, the “excitation/inhibition imbalance” working hypothesis may be invalid. Indeed, it relies solely on findings from the Ts65Dn mouse model of DS which has limitations with regards to predictive and translational relevance [43], and there is currently no clinical evidence of enhanced inhibition in individuals with DS. Since human chromosome 21 has approximately 200–300 genes, other pathways including metabolic pathways are likely involved [44]. Future trials may consider targeting more than one pathway at a time to maximize therapeutic potential.

## Conclusions

Here we have described some of the challenges, and potential strategies to address them, from the perspective of investigators experienced with research in this population [45]. The low drop-out rate of around 9% illustrates the high dedication and motivation from the study participants and their caregivers. Standardization of scale administrations combined with high-quality and consistent training among the different sites and countries allowed us to achieve overall good quality of the data collected with moderate-to-low variability, consistent with what has been previously reported for DS or other conditions with intellectual disability. Independent of the negative outcome of the Clematis study, the learnings on outcome measures and feasibility of conducting international trials in DS, advocacy group relationships, and health authorities’ interactions, provide key information to support future clinical trials in DS and other populations with intellectual disabilities.

## Supplementary Information

**Additional file 1.** Previous clinical information. Summary of PET and MAD data from Study BP25611 and Study BP25543.

**Additional file 2.** Study design.

**Additional file 3.** Primary and secondary assessment scales. Provides more detailed information on the scales, including the DS-CGI-I.

**Additional file 4.** EEG supplementary methods. Provides detailed methodology.

**Additional file 5.** Percent of Participants with Relevant Improvements for each Assessment of the Composite Endpoint by Age Group at 3 and 6 months. A table showing percent of participants with above-threshold improvements for each assessment by age group and time point.

**Additional file 6.** VABS-II: Change from baseline at 6 months. Figure showing VABS-II data: composite and individual scores for socialization, communication, and daily living skills.

**Additional file 7.** Quantitative EEG. Figures showing further analysis of EEG to support the EEG data in the main manuscript.

**Additional file 8 **Estimated Receptor Occupancy and Pharmacokinetics: **Table 1**: Estimated Receptor Occupancy from Geomean Trough Basmisanil Plasma Concentrations (ng/mL) by Age Group and Dose. Table showing trough concentrations and estimated receptor occupancy by age group and dose. **Table 2**: Geomean Trough Basmisanil Plasma Concentration (ng/mL) and Receptor Occupancy by dose. Table showing trough concentrations and estimated receptor occupancy by dose. **Table 3**: Geomean Trough Basmisanil Plasma Concentrations (ng/mL) in Adolescents and Adults by visit and dose. Table showing trough concentrations by age group, dose, and timepoint.

**Additional file 9.** Diastolic and systolic blood pressure. Table summarizing the change from baseline data at 2 weeks, 3 months and 6 months.

**Additional file 10.** ECG QTcF changes from baseline. Table summarizing the change from baseline at 2 weeks, 3 months and 6 months.

**Additional file 11.** Co-morbid Symptoms: Change from Baseline at 6months. Table summarizing Conner’s, ADAMS and CSHQ data.

**Additional file 12.** Change from baseline score for each assessment by age group and timepoint. Table summarizing change from baseline score by age group and timepoint.

## Data Availability

The data that support the findings of this study are available on request from the corresponding author [CG]. The data are not publicly available due to them containing information that could compromise research participant privacy/consent.

## Abbreviations

ADAMS: Anxiety, Depression and Mood Abnormalities
ADHD: Attention-deficit/hyperactivity disorder
AE: Adverse event
BID: Twice daily
BRIEF-P: Behavior Rating Inventory of Executive Function Preschool
CELF-4: Clinical Evaluation of Language Fundamentals-version 4
CELF-P: Clinical Evaluation of Language Fundamentals Preschool-2
C-SSRS: Columbia-Suicide Severity Rating Scale
DS: Down syndrome
DS-CGI-I: Down syndrome-specific Clinical Global Impression-Improvement
ECG: Electrocardiogram
EEG: Electroencephalogram
GABA: Gamma-aminobutyric acid
NAM: Negative allosteric modulator
PedsQL: Pediatric Quality of Life Inventory
PET: Positron emission tomography
PK: Pharmacokinetics
RBANS: Repeatable Battery for the Assessment of Neuropsychological Status
VABS-II: Vineland Adaptive Behavior Scales-II
