# Supplementary material for: A randomized, double-blind, placebo-controlled phase II trial to explore the effects of a GABAA-α5 NAM (basmisanil) on intellectual disability associated with Down syndrome
Source: J Neurodev Disord. 2022 Feb 5;14:10. doi: 10.1186/s11689-022-09418-0 (PMC8903644; doi:10.1186/s11689-022-09418-0)
Supplement: Supplementary file 1 — Additional file 1. Previous clinical information. Summary of PET and MAD data from Study BP25611 and Study BP25543. [file 11689_2022_9418_MOESM1_ESM.doc]

**ADDITIONAL FILE 1: PREVIOUS CLINICAL INFORMATION**

**1) Study BP25611 (ClinicalTrials.gov identifier NCT01667367): PET study**

This was a non-randomized, single-blind, placebo-controlled, two treatment, two period, fixed-sequence crossover study. The relationship between basmisanil exposure and occupancy of α5-containing GABA_A_ receptors by basmisanil has been previously characterized in the healthy adult volunteers from this trial (Hipp et al 2021). The aim of the current study was to assess GABA_A_-α5 receptor expression and occupancy in the brains of individuals with Down syndrome and healthy controls following a single oral administration of basmisanil or placebo.

**Summary of Demographic Characteristics of Participants in PET S**tudy

|  | **Healthy volunteer** **(n=9)** | **Down syndrome** **(n=4)** |
| --- | --- | --- |
| **Age (year)** |  |  |
| Mean (SD) | 25.3 (5.6) | 24.0 (4.8) |
| Median | 23.0 | 24.0 |
| Min - Max | 21–39 | 19–29 |
| **Sex** |  |  |
| Male | 7 | 4 |
| Female | 2 | 0 |
| **Race** |  |  |
| Asian | 2 | 0 |
| Black or African American | 1 | 0 |
| White | 6 | 4 |
| **Weight (kg)** |  |  |
| Mean (SD) | 78.58 (8.75) | 78.75 (14.07) |
| Median | 77.80 | 81.85 |
| Min - Max | 60.2–91.4 | 59.7–91.6 |
| **Height (cm)** |  |  |
| Mean (SD) | 173.67 (11.02) | 156.50 (2.38) |
| Median | 176.0 | 156.5 |
| Min - Max | 153.0–187.0 | 154.0–159.0 |
| **Body Mass Index (kg/m^2^)** | |  |
| Mean (SD) | 26.47 (5.50) | 32.12 (5.51) |
| Median | 24.87 | 33.54 |
| Min - Max | 19.0–36.6 | 24.8–36.6 |

**Treatment**

All participants received placebo and one dose of basmisanil prior to each PET scan. Group sizes are shown in the table below.

|  | **Basmisanil dose** | | | | |
| --- | --- | --- | --- | --- | --- |
|  | **20 mg** | **40 mg** | **80 mg** | **160 mg** | **1000 mg** |
| Healthy volunteers | 2 | 2 | 1 | 2 | 2 |
| Down syndrome | 0 | 1 | 0 | 1 | 2 |

**PET Imaging**

The PET study employed the ^11^C-labelled radioligand Ro 15-4513 to assess brain expression of GABA_A_ receptor subtypes containing an α5 subunit and occupancy of these receptors by basmisanil in adults with Down syndrome and healthy adult volunteers. Participants underwent a PET scan approximately either 3 h (20, 40, 80, 160 mg or placebo) or 5 h (1000 mg or placebo) post dose to coincide with the anticipated time of maximum plasma concentrations of basmisanil. PET scans and analysis were undertaken as described previously (Hipp et al 2021). Blood samples were taken to measure plasma concentrations of basmisanil 30 min pre-PET and post-PET.

PET images were successfully acquired from all sessions. The PET images acquired displayed the expected heterogenous signal, consistent with both the known distribution of GABA_A_ α5 receptors and previous [^11^C]-Ro 15-4513 data. Two of the individuals with Down syndrome requested to abort the PET scanning process before the full 90 minute acquisition was complete (at around 60 and 70 minutes). Subject motion during the scans was also particularly pronounced for one individual with Down syndrome. Software based motion correction was applied to compensate for this, and produced parameter estimates deemed suitable for inclusion in the final data set. However, some motion artefacts remained and may slightly reduce the precision of the resulting parameters. Nevertheless, time-activity curves were generated successfully for all scans.

**Receptor Occupancy Values (%) for a Subset of Specified Regions of Interest**

|  | **Dose** **(mg)** | **Amygdala** | **Hippo** **campus** | **Insular** **cortex** | **Anterior** **cingulate** | **Accumbens** |
| --- | --- | --- | --- | --- | --- | --- |
| **Healthy volunteers** | | | | | | |
| 13040 | **20** | 30.9 | 26.8 | 26.7 | 26.2 | 29.6 |
| 13041 | **20** | 34.7 | 35.8 | 31.7 | 31.7 | 31.8 |
| 11010 | **40** | 53.5 | 48.4 | 44.6 | 44.8 | 52.1 |
| 13010 | **40** | 40.0 | 41.3 | 32.0 | 32.7 | 37.3 |
| 13050 | **80** | 63.5 | 63.0 | 57.8 | 55.8 | 64.2 |
| 11020 | **160** | 91.4 | 87.2 | 75.2 | 75.6 | 86.6 |
| 11021 | **160** | 85.4 | 85.9 | 77.2 | 76.4 | 87.0 |
| 13030 | **1000** | 93.1 | 94.2 | 79.9 | 79.1 | 89.5 |
| 13031 | **1000** | 97.4 | 94.9 | 82.2 | 82.1 | 93.7 |
| **Down syndrome** | | | | | | |
| 14010 | **40** | 57.4 | 55.5 | 49.3 | 48.0 | 52.6 |
| 12020 | **160** | 79.9 | 84.1 | 71.0 | 72.9 | 80.3 |
| 14030 | **1000** | 91.9 | 93.5 | 81.6 | 83.5 | 90.9 |
| 14031 | **1000** | 96.0 | 96.3 | 80.0 | 80.4 | 93.0 |

**Outcome**

- Single doses of RO5186582 up to 1000 mg were tolerated as well as placebo by adults with Down syndrome and healthy adult volunteers.
- The overall pattern of α5-containing GABA_A_ receptor expression was similar in adults with Down syndrome and healthy adult volunteers, being highest in the accumbens and insular and anterior cingulate cortices in both populations.
- There was no difference in basmisanil concentration vs. α5-containing GABA_A_ receptor occupancy relationship between adults with Down syndrome and healthy adult volunteers.
- Receptor occupancy by basmisanil was concentration dependent in all of the selected subset of regions of interest (ROIs) (accumbens, amygdala, anterior cingulate, hippocampus, and insular cortex) and the relationship between basmisanil exposure and receptor occupancy was consistent across the ROIs of particular interest.
- There were also no apparent differences in basmisanil exposures between adults with Down syndrome and healthy adult volunteers for each of the doses tested.
- Hence this provides some reassurance that it is reasonable to extrapolate data generated in healthy volunteers to a Down syndrome population for the purposes of dose selection.

**Reference**

Hipp, J.F., Knoflach, F., Comley, R., Ballard, T.M., Honer, M., Basmisanil, a highly selective GABA_A_- α5 negative allosteric modulator: preclinical pharmacology and demonstration of functional target engagement in man. Scientific Reports, 2021; In press.

**2) Study BP25543 (ClinicalTrials.gov identifier NCT01436955): Multiple ascending dose study in young adults with Down syndrome**

This was a multi-center, randomized, double-blind, placebo-controlled, parallel-group study in individuals with Down syndrome (males and females) aged 18 to 30 years, with multiple doses given in ascending order. Participants were administered either placebo or basmisanil at 80 mg, 160 mg or 370 mg, BID, for 5 weeks. Previous studies confirmed that basmisanil was safe and well tolerated after single and multiple administrations in healthy volunteers (Studies BP25129 and WP25366). The aim of this study was to confirm that the safety, tolerability, and PK of basmisanil were similar in the Down syndrome target population compared to healthy volunteers. Exploratory efficacy measurements were also implemented in this trial in order to assess their suitability for the Phase II (Clematis) trial. Since these have not been published and we refer to this information in the main manuscript, we have provided a summary of the exploratory efficacy results below.

**Table 1. Summary of Demographic Characteristics of Participants by Dose Group**

|  | **Placebo** **(n=10)** | **Basmisanil** | | |
| --- | --- | --- | --- | --- |
|  |  | **80 mg (n=8)** | **160 mg (n=8)** | **370 mg (n=8)** |
| **Age (year)** |  |  |  |  |
| Mean (SD) | 22.8 (4.13) | 22.9 (4.16) | 23.8 (3.85) | 22.9 (3.52) |
| Median | 21.0 | 20.5 | 22.5 | 22.5 |
| Min - Max | 18–30 | 19–30 | 18–29 | 18–27 |
| **Sex** |  |  |  |  |
| Male | 5 | 5 | 6 | 5 |
| Female | 5 | 3 | 2 | 3 |
| **Race** |  |  |  |  |
| Asian | 2 |  |  |  |
| Black or African American | 1 |  |  |  |
| Other | 1 |  | 1 |  |
| White | 6 | 8 | 7 | 8 |
| **Weight (kg)** |  |  |  |  |
| Mean (SD) | 65.19 (14.69) | 62.86 (17.36) | 73.63 (18.91) | 73.83 (14.994) |
| Median | 69.40 | 56.30 | 75.15 | 75.90 |
| Min - Max | 41.2–82.7 | 47.8–95.0 | 54.0–106.5 | 56.7–99.6 |
| **Height (cm)** |  |  |  |  |
| Mean (SD) | 151.1 (9.47) | 149.6 (9.44) | 157.3 (9.59) | 160.0 (8.11) |
| Median | 151.5 | 146.0 | 159.5 | 158.0 |
| Min - Max | 140–164 | 141–168 | 140–171 | 149–171 |
| **Body Mass Index (kg/m^2^)** |  |  |  |  |
| Mean (SD) | 28.35 (5.05) | 27.64 (4.51) | 31.52 (5.44) | 28.71 (4.62) |
| Median | 29.22 | 25.71 | 32.12 | 27.01 |
| Min - Max | 21.02–36.82 | 23.05–37.11 | 24.65–40.19 | 22.71–35.34 |

**EXPLORATORY EFFICACY RESULTS**

**1. Clinical Global Impression (CGI-I)**

CGI-I was assessed at the last treatment day (Day 35-38) and compared to the baseline day (initiation of treatment) using a 7-point scale, i.e., 2 = much improved, 3 = minimally improved, 4 = no change from baseline. Only two participants were minimally improved in the placebo group. In contrast, six participants in the 80 mg group, five participants in the 160 mg group, and three participants in the 370 mg group were either minimally or much improved.

**2. Cambridge Neuropsychological Test Automated Battery (CANTAB)**

**Table 2** summarizes the results of the CANTAB assessments: reaction time (RTI), and paired associates learning (PAL). No relevant differences were seen between the treatment groups for the PAL first trial memory score, the number of patterns reached and the PAL total number of errors. The RTI test revealed that there was some improvement (although not clearly dose-related) in mean values of 5-choice movement times, 5-choice reaction times, and simple movement time for all doses of basmisanil; no improvement was seen with placebo. For simple reaction time, some improvements were seen for the 80 mg and 160 mg basmisanil groups; no improvements were seen for the 370 mg dose or for placebo.

**Table 2. Cambridge Neurop**sychological Test Automated Battery (CANTAB) Assessments by Treatment and Visit

| **Mean (SD)** | **Placebo** | **Basmisanil** | | |
| --- | --- | --- | --- | --- |
|  | **(n=10)** | **80 mg (n=8)** | **160 mg (n=8)** | **370 mg (n=7)** |
| **PAL: 1^st^ trial memory score** | |  |  |  |
| Baseline | 2 (2.2) | 10 (4.7) | 8 (4.5) | 9 (5.3) |
| Day 35-38 | 5 (5.3)^a^ | 10 (5.3) | 9 (6.4) | 9 (5.6)^b^ |
| Change from Baseline | 1 (4.6)^a^ | 0 (3.0) | 0 (2.9) | -1 (4.2)^b^ |
| **PAL: Number of patterns reached** | |  |  |  |
| Baseline | 4 (1.8) | 5 (1.4) | 5 (1.4) | 5 (1.9) |
| Day 35-38 | 4 (1.9)^a^ | 5 (1.4) | 5 (1.6) | 5 (1.7)^b^ |
| Change from Baseline | 0 (0.6)^a^ | 0 (0.4) | 0 (0.5) | 0 (0)^b^ |
| **PAL: Total errors adjusted** | |  |  |  |
| Baseline | 75 (30.8) | 27 (32.1) | 44 (30.7) | 36 (39.6) |
| Day 35-38 | 67 (39.9)^a^ | 29 (31.9) | 37 (36.0) | 34 (40.3)^b^ |
| Change from Baseline | -1 (13.0)^a^ | 3 (14.7) | 3 (8.5) | -3 (10.8)^b^ |
| **RTI: 5-choice movement time (ms)** | |  |  |  |
| Baseline | 577 (149.7) | 659 (226.7) | 609 (246.8) | 604 (226.8) |
| Day 35-38 | 603 (153.3)^b^ | 636 (211.3) | 534 (192.8) | 593 (222.2)^b^ |
| Change from Baseline | 34 (160.8)^b^ | **-101 (204.8)** | **-79 (74.6)** | **-12 (65.3)^b^** |
| **RTI: 5-choice reaction time (ms)** | |  |  |  |
| Baseline | 404 (83.8) | 374 (99.1) | 413 (114.3) | 391 (76.2) |
| Day 35-38 | 391 (127.0)^b^ | 395 (92.7) | 413 (119.2) | 375 (73.9)^b^ |
| Change from Baseline | 49 (95.2)^b^ | **-11 (60.2)** | **-21 (20.4)** | **-6 (53.2)^b^** |
| **RTI: Simple movement time (ms)** | |  |  |  |
| Baseline | 593 (212.6) | 600 (261.6) | 616 (335.7) | 598 (233.7) |
| Day 35-38 | 667 (246.3)^b^ | 623 (248.0) | 563 (246.9) | 599 (196.6)^b^ |
| Change from Baseline | 125 (223.0)^b^ | **-110 (142.1)** | **-73 (128.6)** | **-33 (114.5)^b^** |
| **RTI: Simple reaction time (ms)** | |  |  |  |
| Baseline | 438 (111.3) | 368 (99.9) | 390 (99.6) | 385 (111.3) |
| Day 35-38 | 407 (171.7)^b^ | 371 (88.6) | 398 (129.3) | 368 (82.6)^b^ |
| Change from Baseline | 40 (128.9)^b^ | **-21 (54.3)^a^** | **-34 (54.4)** | 1 (73.1)^b^ |

^a^ n = 8

^b^ n = 7

**3. Vineland-II Adaptive Behavior Scale (VABS-II)**

In terms of VABS-II standard scores (i.e., raw scores standardized for chronological age and for normal population), the biggest positive change (i.e., potential improvement) was observed for the communication domain for the 370 mg dose of basmisanil; no such trends were noted for placebo or lower doses of basmisanil (**Table 3**).

Positive changes from baseline were observed in the socialization domain for the 160 mg dose, but not for the other treatment groups. No relevant changes were recorded for the daily living skills domain.

Positive changes from baseline in the adaptive behavior composite score were reported for the higher doses of basmisanil, but not for the 80 mg dose and placebo.

**Table 3. Vineland-II Adaptive Behavior Scale (VABS-II) domain (standard scores) by Treatment and Visit**

| **Mean (SD)** | **Placebo** | **Basmisanil** | | |
| --- | --- | --- | --- | --- |
|  | **(n=10)** | **80 mg (n=8)** | **160 mg (n=8)** | **370 mg (n=7)** |
| **VABS Adaptive Behavior Composite** | |  |  |  |
| Baseline | 54.6 (13.13) | 57.5 (18.72) | 53.9 (16.38) | 52.5 (16.27) |
| Day 35-38 | 56.3 (8.12)^a^ | 56.5 (19.30) | 55.3 (12.43) | 53.0 (17.13)^b^ |
| Change from Baseline | -1.3 (5.70)^a^ | -1.0 (3.21) | **1.4 (7.61)** | **1.4 (4.76)^b^** |
| **VABS Communication** | |  |  |  |
| Baseline | 52.6 (15.25) | 56.9 (20.81) | 49.3 (21.71) | 44.9 (19.47) |
| Day 35-38 | 51.9 (12.48)^a^ | 54.6 (22.66) | 46.6 (20.42) | 51.9 (16.93)^b^ |
| Change from Baseline | -3.3 (8.92)^a^ | -2.3 (5.20) | -2.6 (13.63) | **5.7 (12.94)^b^** |
| **VABS Daily Living Skills** | |  |  |  |
| Baseline | 53.9 (12.76) | 56.8 (15.06) | 57.8 (14.99) | 57.9 (13.78) |
| Day 35-38 | 58.4 (7.11)^a^ | 57.0 (15.95) | 58.8 (10.02) | 55.1 (13.22)^b^ |
| Change from Baseline | 0.9 (4.16)^a^ | 0.3 (2.92) | 1.0 (7.71) | -1.0 (3.00)^b^ |
| **VABS Socialization** | |  |  |  |
| Baseline | 61.6 (12.45) | 64.1 (22.79) | 61.5 (15.18) | 60.4 (23.03) |
| Day 35-38 | 61.1 (9.66)^a^ | 64.6 (21.84) | 65.0 (15.88) | 58.6 (23.77)^b^ |
| Change from Baseline | -1.8 (7.91)^a^ | 0.5 (3.96) | **3.5 (14.35)** | 0.6 (4.86)^b^ |

^a^ n = 8

^b^ n = 7

**4. Observer Memory Questionnaire-Parent Form (OMQ-PF)**

A marked improvement in the OMQ-PF mean overall score was noted at the end of treatment with 160 mg basmisanil (**Table 4**). No such improvement was noted for any of the other treatment groups. The baseline score for the 160 mg dose group was lower than the other treatment groups, which may have accounted for this marked improvement.

Table 4. Observer Memory Questionnaire-Parent Form (OMQ-PF) total score by Treatment and Visit

| **Mean (SD)** | **Placebo** | **Basmisanil** | | |
| --- | --- | --- | --- | --- |
|  | **(n=10)** | **80 mg (n=8)** | **160 mg (n=8)** | **370 mg (n=7)** |
| **OMQ-PF Total Score** | |  |  |  |
| Baseline | 92.0 (18.57) | 95.4 (14.74) | 86.1 (16.27) | 94.3 (16.79) |
| Day 35-38 | 97.9 (21.67)^a^ | 95.0 (13.83) | 96.0 (16.86) | 91.8 (17.08)^b^ |
| Change from Baseline | 2.6 (7.04)^a^ | -0.4 (4.86) | **9.9 (7.90)** | 0.5 (6.72)^b^ |

^a^ n = 7

^b^ n = 6

**5. Repeatable Battery for the Assessment of Neuropsychological Status (RBANS)**

The RBANS battery to measure cognitive performance was composed of the following tasks: digit span (worst score 0; best score 16), list learning (worst score 0; best score 40), list recall (worst score 0; best score 10), list recognition (worst score 0; best score 20), picture naming (worst score 0; best score 10), semantic fluency (worst score 0; best score 40), and story memory (worst score 0; best score 24).

There was some mean improvement in list learning, list recall and list recognition in participants who received basmisanil and not in those who received placebo (**Table 5**). The 160 mg dose group showed the greatest improvement across all three list tasks. Treatment with basmisanil had no consistent effect on digit span, picture naming, semantic fluency, or story memory (data not shown).

**Table 5. Frequency (%) and Summary Statistics of Repeatable Battery for the Assessment of Neuropsychological Status (RBANS)**

| **RBANS Value**  Change from baseline | **Placebo** | **Basmisanil** | | |
| --- | --- | --- | --- | --- |
|  | **(n=10)** | **80 mg (n=8)** | **160 mg (n=8)** | **370 mg (n=7)** |
|  | **n (%)** | **n (%)** | **n (%)** | **n (%)** |
| **RBANS – List Learning** |  |  |  |  |
| ≤ 0^a^ | 4 (50%) | 5 (63%) | 3 (38%) | 4 (57%) |
| ≥ 1^b^ | 4 (50%) | 3 (38%) | 5 (63%) | 3 (43%) |
| ≥ 2^c^ | 3 (38%) | 3 (38%) | 4 (50%) | 3 (43%) |
| Mean (SD) | -1.5 (5.9) | 0.1 (5.4) | **3.5 (8.9)** | 0.9 (4.3) |
| **RBANS – List Recall** |  |  |  |  |
| ≤ 0^a^ | 7 (88%) | 4 (50%) | 4 (50%) | 4 (57%) |
| ≥ 1^b^ | 1 (13%) | 4 (50%) | 4 (50%) | 3 (43%) |
| ≥ 2^c^ | 1 (13%) | 2 (25%) | 4 (50%) | 1 (14%) |
| Mean (SD) | -1.0 (3.2) | 1.3 (2.7) | **2.1 (3.4)** | -0.3 (2.1) |
| **RBANS – List Recognition** |  |  |  |  |
| ≤ 0^a^ | 7 (88%) | 7 (88%) | 5 (63%) | 6 (86%) |
| ≥ 1^b^ | 1 (13%) | 1 (13%) | 3 (38%) | 1 (14%) |
| ≥ 2^c^ | 0 | 1 (13%) | 2 (25%) | 1 (14%) |
| Mean (SD) | -2.0 (3.6) | -0.1 (1.5) | **1.4 (3.6)** | 0.0 (1.0) |

The highest mean change is shown in bold. SD = standard deviation

^a^ A decrease or no change in RBANS value from baseline.

^b^ An increase in RBANS value from baseline greater than or equal to 1.

^c^ An increase in RBANS value from baseline greater than or equal to 2.

**6. Clinical Evaluation of Language Fundamentals (CELF-2 and CELF-4)**

Participants were given the CELF preschool 2 version and the CELF-4 version for world classes 1 (first level of difficulty) and word classes 2 (second level of difficulty). In both the CELF-2 and CELF-4 tests, higher scores were recorded at baseline and at day 35-38 for receptive tasks compared with expressive tasks, confirming that the receptive task was easier than the expressive task for young adults with Down syndrome. Similarly higher scores were recorded for the CELF-4 word class 1 test compared with the word class 2 test.

There were no noteworthy differences between the basmisanil dose groups or placebo for the CELF-2 expressive or receptive scores. No reliable conclusions can be drawn from the CELF results as the variability was high and the sample population was small in some instances.

**Overall Outcome**

Basmisanil was well tolerated in young adults with Down syndrome after dosing of 80 mg, 160 mg and 370 mg BID for 5 weeks. No drug-related safety pattern emerged. Steady state basmisanil exposures increased in an approximately dose-proportional manner between 80 mg and 160 mg, but exposures in the 370 mg group were similar to those in the 160 mg group.

Positive trends were observed in change from baseline scores when comparing with the placebo group for some cognitive scales (e.g., CANTAB RTI, RBANS list learning, OMQ-PF) and CGI. However, care should be considered while interpreting these results as these assessments were exploratory in nature and the sample size was small.
