# Supplementary material for: A randomized, double-blind, placebo-controlled phase II trial to explore the effects of a GABAA-α5 NAM (basmisanil) on intellectual disability associated with Down syndrome
Source: J Neurodev Disord. 2022 Feb 5;14:10. doi: 10.1186/s11689-022-09418-0 (PMC8903644; doi:10.1186/s11689-022-09418-0)
Supplement: Supplementary file 2 — Additional file 2. Study design. [file 11689_2022_9418_MOESM2_ESM.doc]

**Additional file 2. Study design**

BID = twice daily; taken orally in the morning and evening within 30 min of a meal.

EOT = end of treatment

^a^ Dosage for participants that were 12–13 years of age at the time of randomization.

The study was conducted at 30 sites across 9 countries as follows: US (8 sites), Spain (6 sites), France (4 sites), Mexico (3 sites), Italy (3 sites), New Zealand (3 sites), the UK (1 site), Argentina (1 site) and Canada (1 site).

This was a double-blind study meaning that the treatment allocation was masked to the participants and their carers, to any personnel at the study center as well as Roche study team members (with the exception of the project statistician).

Randomization was performed by the Roche study team using an interactive voice or web response system to generate assigned subject randomization numbers. The randomization numbers were allocated sequentially in the order in which the subjects were enrolled (after the end of the baseline visit).
