# Supplementary material for: A randomized, double-blind, placebo-controlled phase II trial to explore the effects of a GABAA-α5 NAM (basmisanil) on intellectual disability associated with Down syndrome
Source: J Neurodev Disord. 2022 Feb 5;14:10. doi: 10.1186/s11689-022-09418-0 (PMC8903644; doi:10.1186/s11689-022-09418-0)
Supplement: Supplementary file 3 — Additional file 3. Primary and secondary assessment scales. Provides more detailed information on the scales, including the DS-CGI-I. [file 11689_2022_9418_MOESM3_ESM.doc]

**Additional file 3: Primary and Secondary Assessment Scales**

***Repeatable Battery for the Assessment of Neuropsychological Status (RBANS):*** The RBANS was developed for the dual purpose of identifying and characterizing cognitive decline in the older adult and as a neuropsychological screening battery for younger patients (Randolph et al 1998) and can be used to measure cognitive decline or improvement. List learning, list recognition and list recall tasks are subtests of RBANS and were used to assess immediate and delayed verbal memory, as well as language capacities.

***Vineland Adaptive Behavior Scale (Second Edition; Vineland™-II; VABS-II):*** The VABS-II measures adaptive skills such as communication, daily living and socialization skills and provided a composite standard score reflecting an individual’s overall function. The survey interview form was used and administered to parents or caregivers using a semi-structured interview format (Sparrow et al 2005).

***Clinical Global Impression - Improvement (CGI-I):*** The CGI-I is a 7-point scale that requires the clinician to assess how much the participant's illness has improved or worsened relative to a baseline state at the beginning of the intervention, and rated from 1, very much improved; to 7, very much worse. However, the classical CGI rating may be too general to provide meaningful information about patient status or treatment response. To address these challenges, based on a Down syndrome disease conceptual model (Rofail et al 2015a) we aimed to develop a novel, standardized CGI scoring method and a process of training clinicians for use in Phase II and Phase III clinical trials with Down syndrome populations (Rofail et al 2015b). **Six domains were selected and included in the DS-CGI:** Communication/Speech; Activities of Daily Living; Social Functioning and Appropriateness; Stubbornness/Noncompliance; Other Abnormal Behavior; Parent’s Major Concern. It was strongly recommended that the clinician who completed the CGI was not involved in any other cognitive assessments or should have completed the CGI before performing any other cognitive tests or interviews.

***Clinical Evaluation of Language Fundamentals - version 4 (CELF-4):*** Among the subtests available in the CELF, the Word Classes subtest was used to evaluate the ability to understand and express relationships between words that are related by semantic class relationships. The participants were asked to choose the items that best represented the desired relationship then express the relationship. This subtest gives information about the participant’s development of categorization skills and ability to associate word meanings. During the study, if the participant was able to perform the Word Classes 1 without any zero scores on the receptive part of 7 consecutive items (i.e., reaching the ceiling of performance on the test), then the Word Classes 2 (higher difficulty) of the CELF-4 version were administered.

***Behavior Rating Inventory of Executive Function® Preschool (BRIEF-P):*** The BRIEF-P (Gioia et al 2000) is a widely used caregiver questionnaire of everyday skills reflective of abilities in the executive domain. The test-retest reliability has been found to be adequate to high for the parent form (Edgin et al 2010). The global executive composite score was derived for the following domains: inhibit, shift, emotional control, initiate, working memory, plan/organize, organization of materials and monitor.

***Pediatric Quality of Life Inventory^TM^ (PedsQL^TM^):*** The PedsQL measurement model was designed to integrate the merits of generic and disease-specific instruments (Varni et al 1999). Caregivers were asked to start with the cognitive functioning scale module, then the generic core module (version 4) and finally the family impact module (version 2) and a global score was obtained.

**References**

Edgin JO, Mason GM, Allman MJ, et al. Development and validation of the Arizona Cognitive Test Battery for Down syndrome. J Neurodevelop Disord. 2010;2:149-164.

Gioia GA, Isquith PK, Guy SC, Kenworthy L. Behavior rating inventory of executive function. Child Neuropsychol. 2000; 6:235-8.

Randolph C, Tierney MC, Mohr E, Chase TN. The Repeatable Battery for the Assessment of Neuropsychological Status (RBANS): preliminary clinical validity. J Clin Exp Neuropsychol. 1998;20:310.

Rofail D, Marshall, Staunton, Khwaja O, Liogier d'Ardhuy X, Noeldeke J, Abetz-Webb L, Froggatt D, Buckley, S. # WDSC 2015a. Understanding the experiences of living with Down syndrome: the results of in-depth semi-structured interviews with caregivers, teachers, and clinicians.

Rofail D, Marshall, Staunton, Khwaja O, Liogier d'Ardhuy X, Noeldeke, J Abetz-Webb L, Froggatt D, Buckley S # WDSC 2015b. Applying cognitive debriefing methods to assessments used in Down syndrome studies.

Sparrow SS, Cicchetti, DV, Balla, DA. Vineland Adaptive Behavior Scales – second edition. San Antonio: The Psychological Corporation; 2005.

Varni JW, Seid M, Rode CA. The PedsQL: measurement model for the pediatric quality of life inventory. Med Care. 1999;37:126–39.
