# Supplementary material for: A randomized, double-blind, placebo-controlled phase II trial to explore the effects of a GABAA-α5 NAM (basmisanil) on intellectual disability associated with Down syndrome
Source: J Neurodev Disord. 2022 Feb 5;14:10. doi: 10.1186/s11689-022-09418-0 (PMC8903644; doi:10.1186/s11689-022-09418-0)
Supplement: Supplementary file 4 — Additional file 4. EEG supplementary methods. Provides detailed methodology. [file 11689_2022_9418_MOESM4_ESM.doc]

**Additional file 4: EEG supplementary methods**

Initially, participants were not instructed on how to behave during the EEG assessments, causing poor quality EEG data with profound movement artifacts and unclear eyes open / eyes closed status. To improve EEG signal quality for quantitative analyses, the protocol was amended on the 27.4.2015 to instruct participants to have 3 x 2 minutes eyes closed and 3 x 2 minutes eyes open, while trying to stay calm and minimize movements. Investigators were instructed to indicate the beginning and end of these sections via button-presses recorded along with the data by the EEG system and to note down these time points in the transmittal form.

***EEG recording.*** EEG was recorded from 19 Ag/AgCl electrodes arranged according to the 10/20 system (Fp1, Fp2, F7, F3, Fz, F4, F8, T3, C3, Cz, C4, T4, P7, P3, Pz, P4, P8, O1, O2) mounted in an elastic cap (Wavegard, ANT, Netherlands), and attached to the skin with conductive gel (Cleargel Onestep, H + H Medizinprodukte, Germany). EEG was recorded with a sampling rate of 400 Hz (Grass Comet amplifier, Natus Medical Incorporated, Pleasanton, CA, USA; high-pass: 0.3 Hz; low-pass filter: 70 Hz; line-noise notch filter at 60 Hz in US and Mexico sites, 50 Hz all other sites; recording reference Oz).

***Pre-processing and data quality.*** Participants’ data were only used for quantitative analyses if they were consistently instructed or consistently not instruction for the EEG of the conditions to compare. Participants with insufficient EEG data quality (excessive artifacts that prevent a qEEG analysis) or only eyes closed data were excluded (n=15). In total 70 participants provided data for at least one experimental contrast (placebo vs drug, low vs high dose, or week 2 vs week 20); four of which withdrew from the trial but had usable EEG data. These data came from 17 different clinical sites with 4.1 ± 4.4 recordings per site (range 1 to 19). The table below details the number of usable datasets for the different treatment groups and assessment time-points.

**Number of pa**rticipants with valid EEG for the different treatment groups and assessment time points.

|  | **Baseline** | **Week 2** | **Week 20** |
| --- | --- | --- | --- |
| **Placebo** | 19 | 22 (19) | 18 (15) |
| **Low dose** | 21 | 21 (19) | 19 (17) |
| **High dose** | 24 | 25 (24) | 14 (13) |

In parenthesis is the number of participants with valid baseline EEG.

All pre-processing was performed blinded to the treatment group. The analyses were performed on eyes-open data. Eyes-closed data was not analyzed since many participants did not comply with closing their eyes or were not instructed to close their eyes. For participants that were not instructed, sections that resembled calm eyes-open state, i.e. relatively low alpha-band activity, presence of occasional eye blinks, and relatively mild movement artifacts were selected from the continuous data. For instructed participants the validity of markers indicating eyes open sections was confirmed (using eye-blink activity and alpha-band power as a reference signal), and, if necessary, corrected. Channels with excessive noise were identified and interpolated (spline-interpolation using 10/20 standard locations, # interpolated channels per participant: 0.7 ± 0.97, mean ± std, range: 0-4 channels). Channels were interpolated always for all EEG sessions of a participant. Then, eyes open data sections were extracted, band-pass filtered between 1 and 32 Hz (FIR filter, n = 2 x sampling rate), and re-referenced to the average across all electrodes.

Data sections confounded by large technical artifacts (e.g. large-amplitude transients due to changes in impedance) or prominent physiological artifacts (e.g. strong muscle activity) where identified by visual inspection and excluded from further processing. Then independent component analysis (ICA [Jung et al., 2000]; algorithm: FastICA [Hyvarinen, 1999]) was applied to discard remaining artifacts by removing artifactual components (muscle artifacts, ECG artifacts, eye movements and blinks, technical artifacts, n = 4.4 ± 1.40 components per EEG recording, mean ± std, range: 0-8 components).

Pre-processing resulted in 173 ± 79.5 s (mean ± std, range: 66.5 – 428.5 s) of data per participant and condition for analysis. Overall the data quality was considered suitable for the planned analyses as judged by fraction of data rejected as artifactual and qualitative impression of EEG traces.

***Power spectrum.*** Power spectral estimates were derived for logarithmically scaled frequencies with a logarithmic frequency smoothing using Morlet Wavelets (Tallon-Baudry et al., 1997) with a spectral band-width of 1/2 octave (f/σf = 5.83). Center frequencies were spaced logarithmically according to the exponentiation of the base 2 with exponents ranging from 1 (2 Hz) to 5 (32 Hz) in steps of 1/8. Spectral estimates were derived in successive 3/4-overlapping temporal windows. This frequency transform accounts for the logarithmic nature of electrophysiological data (Buzsáki & Draguhn, 2004). For some analyses power was then normalized in the frequency range of 2-32 Hz for each participant and condition (relative power) and re-scaled to the average power across all participants and conditions.

For statistical analyses power values were log-transformed to render distributions more normal. Finally, age and age2 were regressed out from contrasts of interest (placebo vs drug, week 2 vs week 20 for drug, low drug vs high drug) and the reduced degrees of freedom were accounted for in subsequent statistical tests. For illustration log-transformed values were back-transformed (including differences, standard deviations and errors, and confidence intervals).

***Statistics.*** Student's t-tests were used for single comparisons. To test for differences in spectral power across the space of electrodes and frequencies cluster randomization tests were employed (Nichols and Holmes 2002). To this end the group labels for the contrast of interest (e.g. placebo and drug) were randomized (n=10,000), t-test were performed at each electrode and for each frequency, thresholded at p=0.05 (two-tailed), then cluster were identified (contiguous above-threshold values in electrode-frequency space), and the size of the largest cluster across the entire space from each randomization was used to generate a Null-hypothesis distribution. Cluster-randomization statistics accounts for multiple-comparison across all frequencies and electrodes as well as positive and negative changes (two-tailed) in a data-adaptive manner.

Test-retest reliability: ICC analysis (Interclass correlation coefficient type 1-1, [McGraw and 1996]) was applied to the data of all participants in the placebo group that completed all three EEG assessments (n = 15, log-transformed power values for baseline, week 2, and week 20) to quantify the reliability of the spectral measures. On average, across all electrodes and frequencies analyzed, the ICC was 0.74 ± 0.125 (**see Additional file 7**). This magnitude is in line with previous reports on the reliability of power spectral EEG features (Gudmundsson et al., 2007) and is considered sufficient for quantitative analysis.

The raw EEG traces were also reviewed by a blinded experienced neurologist to evaluate presence of epileptic activity.

**References**

Buzsáki, G., & Draguhn, A. (2004). Neuronal oscillations in cortical networks. Science, 304(5679), 1926-1929. doi:10.1126/science.1099745

Hyvarinen, A. (1999). Fast and robust fixed-point algorithms for independent component analysis. IEEE Trans. Neural Netw. 10, 626–634.

Jung, T.-P., Makeig, S., Westerfield, M., Townsend, J., Courchesne, E., and Sejnowski, T.J. (2000). Removal of eye activity artifacts from visual event-related potentials in normal and clinical subjects. Clin. Neurophysiol. 111, 1745–1758.

McGraw, K.O., and P, S. (1996). Forming inferences about some intraclass correlation coefficients. Psychol. Methods 1, 30–46.

Nichols, T.E., and Holmes, A.P. (2002). Nonparametric permutation tests for functional neuroimaging: A primer with examples. Hum. Brain Mapp. 15, 1–25.

Rofail D, Marshall, Staunton, Khwaja O, Liogier d'Ardhuy X, Noeldeke, J Abetz-Webb L, Froggatt D, Buckley S # WDSC 2015 Applying cognitive debriefing methods to assessments used in Down syndrome studies.

Tallon-Baudry, C., Bertrand, O., Delpuech, C., and Pernier, J. (1997). Oscillatory gamma-Band (30–70 Hz) Activity Induced by a Visual Search Task in Humans. J. Neurosci. 17, 722–734.
