# Supplementary material for: A randomized, double-blind, placebo-controlled phase II trial to explore the effects of a GABAA-α5 NAM (basmisanil) on intellectual disability associated with Down syndrome
Source: J Neurodev Disord. 2022 Feb 5;14:10. doi: 10.1186/s11689-022-09418-0 (PMC8903644; doi:10.1186/s11689-022-09418-0)
Supplement: Supplementary file 5 — Additional file 5. Percent of Participants with Relevant Improvements for each Assessment of the Composite Endpoint by Age Group at 3 and 6 months. A table showing percent of participants with above-threshold improvements for each assessment by age group and time point. [file 11689_2022_9418_MOESM5_ESM.doc]

**Additional file 5: Percent of Participants with Relevant Improvements for each Assessment of the Composite Endpoint by Age Group at 3 and 6 months**

| **Assessment**  (relevant improvement) | **Age Group**  **(years)** | **Time point (months)** | **Percent of Participants** | | |
| --- | --- | --- | --- | --- | --- |
|  |  |  | **Placebo** | **120 mg (80 mg)** | **240 mg (160 mg)** |
| **RBANS**  **List Learning**  (change ≥ 2 points) | **Combined**  **(12-30)** | 3 | 48.1 | 59.6 | 47.9 |
|  |  | 6 | 60.8 | 53.2 | 52.3 |
|  | **Adolescent**  **(12-17)** | 3 | 42.3 | 64.0 | 38.5 |
|  |  | 6 | 70.8 | 50.0 | 54.2 |
|  | **Adult**  **(18-30)** | 3 | 53.6 | 54.5 | 59.1 |
|  |  | 6 | 51.9 | 56.5 | 50.0 |
| **RBANS**  **List Recall**  (change ≥ 1 point) | **Combined**  **(12-30)** | 3 | 35.8 | 31.9 | 37.5 |
|  |  | 6 | 27.5 | 34.0 | 31.8 |
|  | **Adolescent**  **(12-17)** | 3 | 20.0 | 28.0 | 30.8 |
|  |  | 6 | 20.8 | 33.3 | 33.3 |
|  | **Adult**  **(18-30)** | 3 | 50.0 | 36.4 | 45.5 |
|  |  | 6 | 33.3 | 34.8 | 30.0 |
| **RBANS**  **List Recognition**  (change ≥ 1 points) | **Combined**  **(12-30)** | 3 | 32.1 | 46.8 | 43.8 |
|  |  | 6 | 43.1 | 53.2 | 52.3 |
|  | **Adolescent**  **(12-17)** | 3 | 36.0 | 48.0 | 42.3 |
|  |  | 6 | 37.5 | 50.0 | 54.2 |
|  | **Adult**  **(18-30)** | 3 | 28.6 | 45.5 | 45.5 |
|  |  | 6 | 48.1 | 56.5 | 50.0 |
| **VABS-II**  **Composite Score**  (change ≥ 7 points) | **Combined**  **(12-30)** | 3 | 15.1 | 8.7 | 8.5 |
|  |  | 6 | 18.0 | 8.7 | 16.3 |
|  | **Adolescent**  **(12-17)** | 3 | 23.1 | 0 | 7.7 |
|  |  | 6 | 12.5 | 4.2 | 16.7 |
|  | **Adult**  **(18-30)** | 3 | 7.4 | 19.0 | 9.5 |
|  |  | 6 | 23.1 | 13.6 | 15.8 |
| **DS-CGI-I**  (value ≤ 3) | **Combined**  **(12-30)** | 3 | 53.7 | 38.8 | 32.0 |
|  |  | 6 | 70.0 | 57.4 | 45.7 |
|  | **Adolescent**  **(12-17)** | 3 | 65.4 | 44.0 | 46.2 |
|  |  | 6 | 70.8 | 70.8 | 62.5 |
|  | **Adult**  **(18-30)** | 3 | 42.9 | 33.3 | 16.7 |
|  |  | 6 | 69.2 | 43.5 | 27.3 |

Abbreviations: DS-CGI = Down Syndrome Clinical Global Impression-Improvement; RBANS = Repeatable Battery for the Assessment of Neuropsychological Status; VABS-II = Vineland Adaptive Behavior Scale-II.
