# Supplementary material for: A randomized, double-blind, placebo-controlled phase II trial to explore the effects of a GABAA-α5 NAM (basmisanil) on intellectual disability associated with Down syndrome
Source: J Neurodev Disord. 2022 Feb 5;14:10. doi: 10.1186/s11689-022-09418-0 (PMC8903644; doi:10.1186/s11689-022-09418-0)
Supplement: Supplementary file 6 — Additional file 6. VABS-II: Change from baseline at 6 months. Figure showing VABS-II data: composite and individual scores for socialization, communication, and daily living skills. [file 11689_2022_9418_MOESM6_ESM.doc]

**Additional file 6. VABS-II: Change from baseline at 6 months**

Change from baseline at 6 months of basmisanil treatment and placebo on (**A**) Composite standard score; and (**B**) Individual standard scores for the socialization, communication, and daily living skills domains of the Vineland-II Adaptive Behavior Scale (VABS-II). Error bars indicate standard deviation.
