# Supplementary material for: A randomized, double-blind, placebo-controlled phase II trial to explore the effects of a GABAA-α5 NAM (basmisanil) on intellectual disability associated with Down syndrome
Source: J Neurodev Disord. 2022 Feb 5;14:10. doi: 10.1186/s11689-022-09418-0 (PMC8903644; doi:10.1186/s11689-022-09418-0)
Supplement: Supplementary file 7 — Additional file 7. Quantitative EEG. Figures showing further analysis of EEG to support the EEG data in the main manuscript. [file 11689_2022_9418_MOESM7_ESM.doc]

**Additional file 7. Quantitative EEG.**


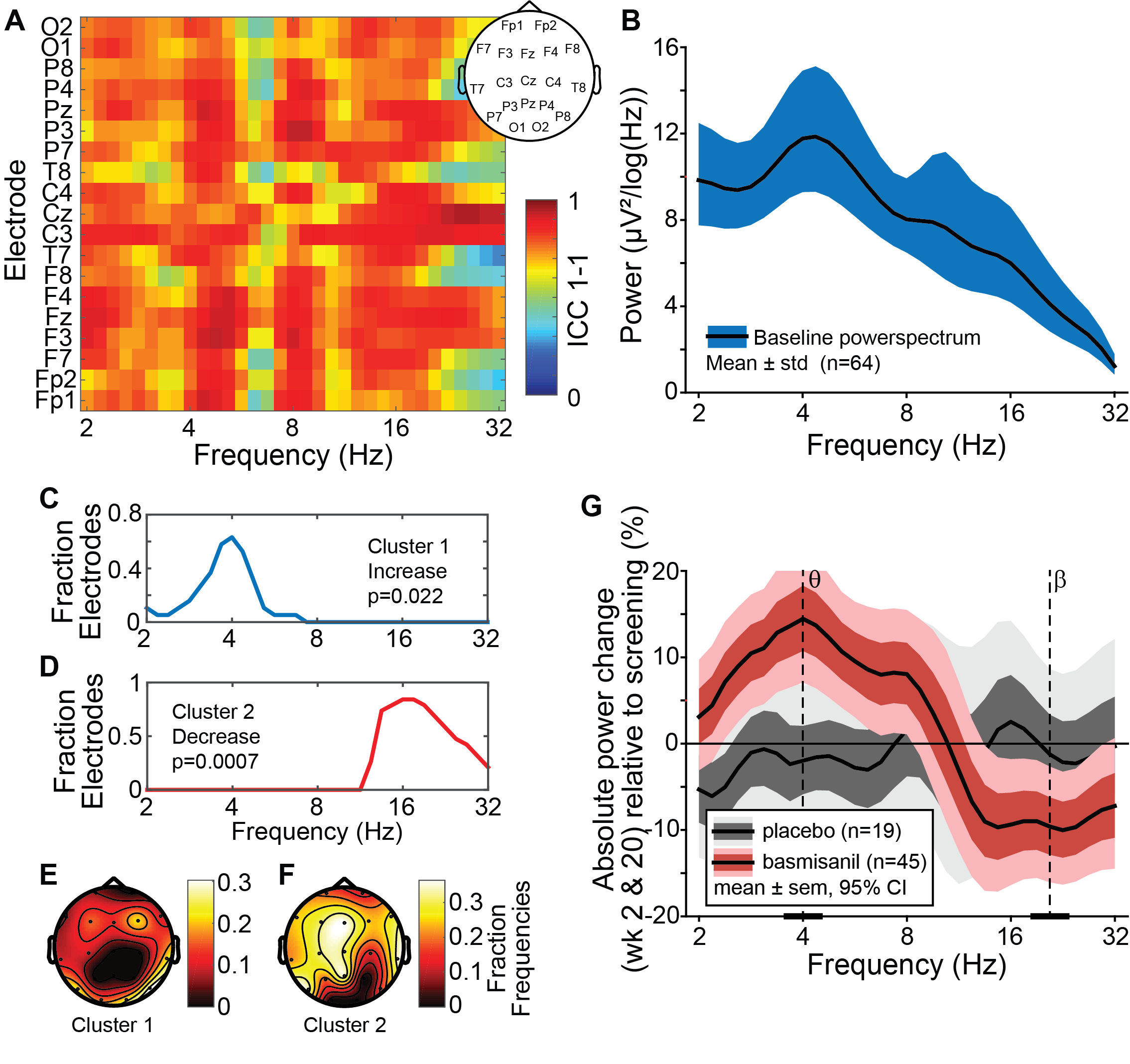


(A) Test-retest reliability (ICC type 1-1 (McGraw and P, 1996)) derived from all participants in the placebo group that had three valid EEG visits (n = 15). (B) Power spectrum at baseline averaged across all electrodes (all participants with EEG). (C-F) Spatial and spectral projections of two significant clusters identified in a cluster-randomization test for differences in EEG signal power (two-tailed) between dosed group (low and high dose groups combined) and placebo group in the frequency range of 2-32 Hz and across all electrodes. (G) Absolute power spectral changes relative to baseline averaged across all electrodes.
