# Supplementary material for: A randomized, double-blind, placebo-controlled phase II trial to explore the effects of a GABAA-α5 NAM (basmisanil) on intellectual disability associated with Down syndrome
Source: J Neurodev Disord. 2022 Feb 5;14:10. doi: 10.1186/s11689-022-09418-0 (PMC8903644; doi:10.1186/s11689-022-09418-0)
Supplement: Supplementary file 8 — Additional file 8 Estimated Receptor Occupancy and Pharmacokinetics: Table 1: Estimated Receptor Occupancy from Geomean Trough Basmisanil Plasma Concentrations (ng/mL) by Age Group and Dose. Table showing trough concentrations and estimated receptor occupancy by age group and dose. Table 2: Geomean Trough Basmisanil Plasma Concentration (ng/mL) and Receptor Occupancy by dose. Table showing trough concentrations and estimated receptor occupancy by dose. Table 3: Geomean Trough Basmisanil Plasma Concentrations (ng/mL) in Adolescents and Adults by visit and dose. Table showing trough concentrations by age group, dose, and timepoint. [file 11689_2022_9418_MOESM8_ESM.doc]

**Additional file 8.**

Table 1: Estimated Receptor Occupancy from Geomean Trough Basmisanil Plasma Concentrations (ng/mL) by Age Group and Dose

|  | **Adolescents** | | | | | | | | | | | | **Adults** | |
| --- | --- | --- | --- | --- | --- | --- | --- | --- | --- | --- | --- | --- | --- | --- |
| **Age group (years)** | **12–13** | | **12–13** | | **14–17** | | **14–17** | | **12-17** | | **12-17** |  | **18–30** | **18–30** |
| **Dose (mg BID)** | **80** | | **160** | | **120** | | **240** | | **80/120** | | **160/240** |  | **120** | **240** |
|  | 24 | | 38 | | 48 | | 38 | | 72 | | 76 |  | 73 | 75 |
| **Basmisanil concentration (ng/mL)** | 1030 | | 1989 | | 1196 | | 2687 | | 1138 | | 2311 |  | 1709 | 2536 |
|  |  |  | |  | |  | |  | |  | |  |  |  |
| **RO% Hippocampus** | 74 | 90 | | 79 | | 93 | | 77 | | 92 | |  | 87 | 93 |
| **N** | 9 | 13 | | 17 | | 14 | | 26 | | 27 | |  | 26 | 26 |

**Table 2: Geomean Trough Basmisanil Plasma Concentration (ng/mL) and Receptor Occupancy (RO)** by dose

|  | **Adolescents & Adults** | |
| --- | --- | --- |
|  | **Low dose**  **80/120mg BID** | **high dose**  **160/240mg BID** |
| **N** | 52 | 53 |
| Plasma Concentration (CV%) | 1396 (60) | 2420 (53) |
| Predicted RO% Hippocampus | 83 | 92 |

Table 3: Geomean Trough Basmisanil Plasma Concentrations (ng/mL) in Adolescents and Adults by visit and dose

|  |  |  | | |  | | |
| --- | --- | --- | --- | --- | --- | --- | --- |
| **Visit** | **Time** | **Low dose**  **120mg/80mg Basmisanil BID** | | | **High dose**  **240mg/160mg Basmisanil BID** | | |
|  |  | **Adolescents** | **Adults** | | **Adolescents** | **Adults** | |
| 2 weeks | Pre-dose | 1284 | 1835 | | 2114 | 2476 | |
|  |  | (n=22) | (n=23) | | (n=27) | (n=25) | |
| 6 weeks | Pre-dose | 984 | 1656 | | 2193 | 2657 | |
|  |  | (n=25) | (n=25) | | (n=25) | (n=26) | |
| 12 weeks | Pre-dose | 1249 | 1674 | | 2438 | 2477 | |
|  |  | (n=25) | | (n=25) | (n=24) | | (n=24) |
