# Supplementary material for: A randomized, double-blind, placebo-controlled phase II trial to explore the effects of a GABAA-α5 NAM (basmisanil) on intellectual disability associated with Down syndrome
Source: J Neurodev Disord. 2022 Feb 5;14:10. doi: 10.1186/s11689-022-09418-0 (PMC8903644; doi:10.1186/s11689-022-09418-0)
Supplement: Supplementary file 9 — Additional file 9. Diastolic and systolic blood pressure. Table summarizing the change from baseline data at 2 weeks, 3 months and 6 months. [file 11689_2022_9418_MOESM9_ESM.doc]

**Additional file 9: Diastolic and Systolic Blood Pressure**

| **Time point** | | **Diastolic Blood Pressure** | | | **Systolic Blood Pressure** | | |
| --- | --- | --- | --- | --- | --- | --- | --- |
|  |  | **Placebo** | **120 mg (80 mg)** | **240 mg (160 mg)** | **Placebo** | **120 mg (80 mg)** | **240 mg (160 mg)** |
| **Baseline**  Mean (SD) | 66.07 (8.4) | | 65.75 (8.9) | 64.44 (9.3) | 112.50 (8.6) | 109.93 (12.6) | 111.74 (12.3) |
| n | | 58 | 55 | 57 | 58 | 55 | 57 |
| **Change from baseline:** | | | | | | | |
| **2 weeks**  Mean (SD) | | -2.05 (9.45) | -0.44 (7.79) | 2.46 (8.45) | -1.12 (9.53) | 1.62 (10.78) | 1.95 (8.79) |
| n | | 57 | 55 | 56 | 57 | 55 | 56 |
| **3 months**  Mean (SD) | | -1.05 (9.75) | -0.68 (8.50) | 0.67 (9.34) | -1.91 (9.79) | 1.00 (8.88) | 0.35 (10.21) |
| n | | 56 | 53 | 52 | 56 | 53 | 52 |
| **6 months**  Mean (SD) | | -0.56 (8.53) | -0.96 (9.20) | 1.1 (10.35) | -2.87 (9.37) | 1.14 (12.10) | 0.24 (12.32) |
| n | | 52 | 51 | 49 | 52 | 51 | 49 |
