# Supplementary material for: A randomized, double-blind, placebo-controlled phase II trial to explore the effects of a GABAA-α5 NAM (basmisanil) on intellectual disability associated with Down syndrome
Source: J Neurodev Disord. 2022 Feb 5;14:10. doi: 10.1186/s11689-022-09418-0 (PMC8903644; doi:10.1186/s11689-022-09418-0)
Supplement: Supplementary file 10 — Additional file 10. ECG QTcF changes from baseline. Table summarizing the change from baseline at 2 weeks, 3 months and 6 months. [file 11689_2022_9418_MOESM10_ESM.doc]

**Additional file 10: ECG QTcF Changes from Baseline**

| **Time point** | **Placebo** | **120 mg (80 mg)** | **240 mg (160 mg)** |
| --- | --- | --- | --- |
| **Baseline**  Mean (SD) | 401.88 (19.28) | 400.80 (20.86) | 399.19 (17.65) |
| n | 58 | 55 | 57 |
| **Change from baseline:** | | | |
| **2 weeks**  Mean (SD) | -0.04 (12.54) | 0.34 (15.22) | 1.13 (13.21) |
| n | 56 | 53 | 55 |
| **3 months**  Mean (SD) | -1.61 (12.34) | 2.92 (13.74) | 0.12 (13.25) |
| n | 56 | 52 | 51 |
| **6 months**  Mean (SD) | 3.42 (14.95) | 2.55 (12.54) | 5.82 (14.26) |
| n | 53 | 51 | 49 |

Parameter: QTcF [msec] (RR: 350–450).

In vitro, basmisanil inhibits hERG channel with an IC_20_ of 624 ng/mL and inhibition of this potassium channel has been linked to QT prolongation. The lack of QTcF prolongation in this study is considered to be in line with C_max,free_ concentrations estimated to be lower than the IC_20_ for hERG channel blockade in vitro.
