# Supplementary material for: A randomized, double-blind, placebo-controlled phase II trial to explore the effects of a GABAA-α5 NAM (basmisanil) on intellectual disability associated with Down syndrome
Source: J Neurodev Disord. 2022 Feb 5;14:10. doi: 10.1186/s11689-022-09418-0 (PMC8903644; doi:10.1186/s11689-022-09418-0)
Supplement: Supplementary file 11 — Additional file 11. Co-morbid Symptoms: Change from Baseline at 6months. Table summarizing Conner’s, ADAMS and CSHQ data. [file 11689_2022_9418_MOESM11_ESM.doc]

**Additional file 11: Co-morbid Symptoms: Change from Baseline at 6 months**

| **Scale** | **Subscale** | **Placebo** | | **120 mg (80 mg)** | | **240 mg (160 mg)** | |
| --- | --- | --- | --- | --- | --- | --- | --- |
|  |  | **Baseline** | **Change** | **Baseline** | **Change** | **Baseline** | **Change** |
| **Conner’s 3^rd^ Edition-Parent Short Form** | Aggression | 0.84 (1.50) | -0.10 (1.27) | 1.27 (1.96) | -0.58 (1.51) | 1.06 (1.69) | 0.07 (1.87) |
|  | Executive functioning | 6.14 (4.02) | -1.59 (2.93) | 5.76 (3.42) | -1.43 (2.46) | 5.65 (3.10) | -1.55 (2.93) |
|  | Hyperactivity | 3.18 (3.34) | -1.33 (2.50) | 3.75 (3.92) | -1.19 (2.95) | 3.00 (3.22) | -0.82 (2.97) |
|  | Inattention | 7.11 (3.57) | -1.16 (3.87) | 6.63 (3.76) | -0.83 (2.98) | 6.77 (3.31) | -1.19 (2.71) |
|  | Learning problem | 8.72 (3.69) | -0.75 (3.24) | 8.68 (3.42) | -1.19 (2.72) | 8.79 (3.12) | -1.57 (3.54) |
|  | Negative impression | 7.11 (2.30) | -0.37 (3.02) | 7.14 (2.16) | -0.90 (2.90) | 6.98 (2.85) | -0.30 (4.08) |
|  | Positive impression | 8.38 (2.39) | -0.63 (2.51) | 8.31 (2.36) | -0.63 (3.34) | 8.36 (2.70) | -0.84 (3.48) |
|  | Peer relation | 4.05 (3.41) | -0.61 (3.01) | 4.24 (3.09) | -0.48 (2.51) | 3.80 (3.23) | -0.64 (3.28) |
| **Anxiety, Depression and Mood Abnormalities (ADAMS)** | Depressed mood | 2.54 (2.86) | -0.22 (2.09) | 2.10 (1.98) | -0.28 (1.53) | 2.50 (3.13) | -0.65(2.28) |
|  | General anxiety | 2.58 (2.29) | -0.62 (1.48) | 2.48 (2.45) | -0.17 (1.96) | 2.63 (2.87) | -0.73 (2.31) |
|  | Manic / hyperactive behavior | 3.23 (2.43) | -0.67 (1.66) | 3.96 (3.12) | -0.79 (2.57) | 3.10 (2.80) | -0.70 (2.26) |
|  | Obsessive / compulsive behavior | 1.86 (2.02) | -0.41 (1.65) | 1.92 (1.83) | -0.45 (1.38) | 1.67 (1.78) | -0.49 (1.56) |
|  | Social avoidance | 3.84 (3.94) | -0.48 (2.56) | 4.26 (3.74) | -0.43 (1.96) | 3.75 (3.86) | -0.72 (2.52) |
| **Children’s Sleep Habit Questionnaire (CSHQ)** | Bedtime scale | 11.53 (3.85) | -0.16 (4.15) | 12.06 (4.07) | -0.38 (3.40) | 11.62 (3.73) | -1.02 (2.75) |
|  | Morning wake-up | 5.51 (2.16) | -0.73 (1.98) | 6.02 (1.94) | -0.63 (2.08) | 5.60 (2.17) | 0.00 (1.66) |
|  | Sleep behavior | 7.89 (2.61) | -0.54 (2.55) | 8.60 (2.75) | -0.96 (1.97) | 7.75 (3.25) | -0.42 (2.39) |
|  | Waking during the night | 1.50 (1.55) | -0.15 (1.07) | 1.61 (1.61) | -0.15 (1.29) | 1.47 (1.56) | 0.30 (1.75) |

Data are presented as: mean (standard deviation).

All questionnaires were used to obtain the parent’s/caregiver’s observations about the participant. Conner’s is designed to assess Attention Deficit/Hyperactivity Disorder (ADHD) and its most common comorbid emotional and behavioral disturbances. ADAMS is designed to assess anxiety, depression and mood disorders in individuals with intellectual disability. CHSQ assesses sleep habits and possible difficulties with sleep.
