# Supplementary material for: A randomized, double-blind, placebo-controlled phase II trial to explore the effects of a GABAA-α5 NAM (basmisanil) on intellectual disability associated with Down syndrome
Source: J Neurodev Disord. 2022 Feb 5;14:10. doi: 10.1186/s11689-022-09418-0 (PMC8903644; doi:10.1186/s11689-022-09418-0)
Supplement: Supplementary file 12 — Additional file 12. Change from baseline score for each assessment by age group and timepoint. Table summarizing change from baseline score by age group and timepoint. [file 11689_2022_9418_MOESM12_ESM.doc]

**Additional file 12. Change from Baseline Score for each Assessment by Age Group at 3 and 6 months**

| **Assessment** | **Age Group** | **Time point** (month) | **Placebo** | | **120 (80) mg** | | **240 (160) mg** | |
| --- | --- | --- | --- | --- | --- | --- | --- | --- |
|  |  |  | **Mean ± SD** | **n** | **Mean ± SD** | **n** | **Mean ± SD** | **n** |
| **Repeatable Battery for the Assessment of Neuropsychological Status (RBANS)** | | | | | | | | |
| **List Learning** | 12-17 | 3 | 0.4 ± 5.6 | 26 | 3.1 ± 5.2 | 25 | -0.4 ± 5.2 | 26 |
|  |  | 6 | 3.3 ± 6.2 | 24 | 2.7 ± 6.4 | 24 | 2.4 ± 5.8 | 24 |
|  | 18-30 | 3 | 2.6 ± 6.6 | 28 | 1.4 ± 5.7 | 22 | 2.6 ± 4.6 | 22 |
|  |  | 6 | 3.0 ± 6.1 | 27 | 2.7 ± 6.0 | 23 | 2.3 ± 3.4 | 20 |
| **List Recall** | 12-17 | 3 | -0.08 ± 2.3 | 25 | -0.08 ± 1.8 | 25 | 0.3 ± 2.8 | 26 |
|  |  | 6 | 0.4 ± 1.7 | 24 | 0.1 ± 2.4 | 24 | -0.08 ± 2.7 | 24 |
|  | 18-30 | 3 | 1.04 ± 3.02 | 28 | 0.4 ± 2.6 | 22 | 0.5 ± 2.2 | 22 |
|  |  | 6 | 0.2 ± 2.9 | 27 | 0.3 ± 3.8 | 23 | -0.1 ± 2.0 | 20 |
| **List Recognition** | 12-17 | 3 | 0.2 ± 2.5 | 25 | 0.9 ± 2.2 | 25 | 0.2 ± 4.4 | 26 |
|  |  | 6 | 1.2 ± 3.1 | 24 | 0.8 ± 3.0 | 24 | 2.2 ± 4.3 | 24 |
|  | 18-30 | 3 | -0.14 ± 3.3 | 28 | 1.3 ± 2.5 | 22 | 1.6 ± 3.9 | 22 |
|  |  | 6 | 1.3 ± 3.5 | 27 | 2.0 ± 3.2 | 23 | 1.3 ± 3.5 | 20 |
| **Vineland Adaptive Behavior Scale-II (VABS-II)** | | | | | | | | |
| **Composite Score** | 12-17 | 3 | 2.7 ± 6.0 | 26 | -0.3 ± 4.1 | 25 | 1.5 ± 3.3 | 26 |
|  |  | 6 | -2.8 ± 7.6 | 24 | -1.0 ± 3.9 | 24 | 2.0 ± 5.0 | 24 |
|  | 18-30 | 3 | 0.5 ± 3.6 | 27 | 2.5 ± 4.9 | 21 | 0.5 ± 4.1 | 21 |
|  |  | 6 | 2.0 ± 12.2 | 26 | 3.1 ± 4.0 | 22 | 2.1 ± 4.1 | 19 |
| **Clinical Evaluation of Language Fundamentals (CELF-4): Word Classes 1** | | | | | | | | |
| **Receptive** | 12-17 | 3 | -0.8 ± 3.8 | 26 | -0.2 ± 3.1 | 25 | 1.04 ± 3.1 | 26 |
|  |  | 6 | 0.5 ± 3.6 | 24 | 0.4 ± 2.8 | 24 | 1.5 ± 3.9 | 24 |
|  | 18-30 | 3 | 0.4 ± 3.5 | 28 | -0.7 ± 5.03 | 22 | 1.1 ± 2.5 | 22 |
|  |  | 6 | 0.9 ± 3.6 | 27 | -0.6 ± 4.0 | 22 | 1.6 ± 3.5 | 20 |
| **Expressive** | 12-17 | 3 | 0.2 ± 4.6 | 26 | 0.4 ± 2.7 | 25 | 1.2 ± 3.7 | 26 |
|  |  | 6 | 0.5 ± 3.4 | 24 | 0.5 ± 2.9 | 24 | 1.3 ± 3.6 | 24 |
|  | 18-30 | 3 | 0.9 ± 2.9 | 28 | 0.9 ± 4.9 | 22 | 1.3 ± 2.4 | 22 |
|  |  | 6 | 1.3 ± 2.9 | 27 | 0.1 ± 4.6 | 22 | 1.2 ± 2.3 | 20 |
| **CELF-4: Word Classes 2** | | | | | | | | |
| **Receptive** | 12-17 | 3 | 0.2 ± 1.5 | 24 | -0.2 ± 1.8 | 22 | 0.00 ± 2.1 | 24 |
|  |  | 6 | 0.3 ± 2.0 | 24 | 0.5 ± 2.2 | 24 | 0.2 ± 2.0 | 24 |
|  | 18-30 | 3 | 0.00 ± 2.5 | 25 | -0.2 ± 2.2 | 21 | 0.00 ± 2.3 | 17 |
|  |  | 6 | 0.8 ± 4.6 | 26 | 0.4 ± 3.8 | 22 | 0.2 ± 2.6 | 21 |
| **Expressive** | 12-17 | 3 | 0.2 ± 1.02 | 24 | 0.05 ± 0.8 | 22 | 0.2 ± 1.4 | 24 |
|  |  | 6 | 0.7 ± 1.9 | 24 | 0.1 ± 1.3 | 24 | 0.2 ± 1.7 | 24 |
|  | 18-30 | 3 | 0.2 ± 1.8 | 27 | -0.2 ± 1.4 | 21 | 0.3 ± 1.6 | 19 |
|  |  | 6 | -0.4 ± 2.7 | 25 | -0.2 ± 1.6 | 21 | 0.2 ± 1.6 | 17 |
| **Behavior Rating Inventory of Executive Function Preschool (BRIEF-P)*** | | | | | | | | |
| **Global Executive Score** | 12-17 | 3 | -5.9 ± 13.4 | 25 | -7.4 ± 15.4 | 25 | -5.4 ± 10.6 | 26 |
|  |  | 6 | -6.0 ± 11.0 | 24 | -10.9 ± 14.0 | 24 | -8.6 ± 12.2 | 24 |
|  | 18-30 | 3 | -2.5 ± 11.1 | 28 | -5.6 ± 9.0 | 22 | -5.0 ± 13.0 | 22 |
|  |  | 6 | -2.5 ± 13.1 | 27 | -4.6 ± 10.1 | 22 | -6.9 ± 13.6 | 18 |
| **Pediatric Quality of Life Inventory (PedsQL)** | | | | | | | | |
| **Global Score** | 12-17 | 3 | 1.1 ± 12.2 | 26 | 3.1 ± 14.98 | 25 | 2.2 ± 12.02 | 24 |
|  |  | 6 | 2.4 ± 10.0 | 23 | 5.7 ± 12.2 | 24 | 2.4 ± 10.9 | 22 |
|  | 18-30 | 3 | 0.6 ± 15.7 | 28 | 2.1 ± 16.0 | 23 | 5.7 ± 12.2 | 21 |
|  |  | 6 | 1.0 ± 15.0 | 24 | 5.4 ± 13.2 | 22 | 4.9 ± 8.3 | 19 |

*Negative change = improvement; SD = standard deviation.
